# Proteomic response of methicillin-resistant *S. aureus* to a synergistic antibacterial drug combination: a novel erythromycin derivative and oxacillin

**DOI:** 10.1038/srep19841

**Published:** 2016-01-25

**Authors:** Xiaofen Liu, Pei-Jin Pai, Weipeng Zhang, Yingwei Hu, Xiaojing Dong, Pei-yuan Qian, Daijie Chen, Henry Lam

**Affiliations:** 1 Department of Chemical and Biomolecular Engineering, The Hong Kong University of Science and Technology, Clear Water Bay, Kowloon, Hong Kong; 2 Institute of Antibiotics, Huashan Hospital affiliated to Fudan University, Shanghai, China; 3 Key Laboratory of Clinical Pharmacology of Antibiotics, National Population and Family Planning Commission, Shanghai, China; 4 Graduate School of Medicine, Kaohsiung Medical University, Kaohsiung, Taiwan; 5 Center for Infectious Disease and Cancer Research, Kaohsiung Medical University, Kaohsiung, Taiwan; 6 KAUST Global Partnership program, Division of Life Science, The Hong Kong University of Science and Technology, Clear Water Bay, Kowloon, Hong Kong; 7 State Key Laboratory of New Drug and Pharmaceutical Process, Shanghai Institute ofPharmaceutical Industry, China State Institute of Pharmaceutical Industry, Shanghai, China; 8 School of Pharmacy, Shanghai Jiao Tong University, Shanghai, China; 9 Division of Biomedical Engineering, The Hong Kong University of Science and Technology, Clear Water Bay, Kowloon, Hong Kong

## Abstract

The use of antibacterial drug combinations with synergistic effects is increasingly seen as a critical strategy to combat multi-drug resistant bacteria such as methicillin-resistant *Staphylococcus aureus* (MRSA). In this work, the proteome responses in MRSA under the stress of a sub-inhibitory dose of a synergistic drug combination of a novel erythromycin derivative, SIPI-8294, and oxacillin, were studied by label-free quantitative proteomics. Several control treatment groups were designed to isolate proteome responses potentially related to the synergy: (1) the non-synergistic drug combination of erythromycin and oxacillin, (2) SIPI-8294 only, (3) oxacillin only and (4) erythromycin only. Results showed that 200 proteins were differentially expressed in SIPI-8294/oxacillin-treated cells. Among these proteins, the level of penicillin binding protein 2a, the protein mainly responsible for oxacillin resistance in MRSA, was four times lower in the SIPI-8294/oxacillin group than in the erythromycin/oxacillin group, suggesting that SIPI-8294 may interfere with this known oxacillin resistance mechanism. Moreover, hierarchical clustering analysis of differentially expressed proteins under different treatments revealed that SIPI-8294/oxacillin elicits very different responses than the individual drugs or the non-synergistic erythromycin/oxacillin combination. Bioinformatic analysis indicated that the synergistic effect can be further traced to a disruption in oxidation-reduction homeostasis and cell wall biosynthesis.

*Staphylococcus aureus* (*S. aureus*) can cause various kinds of infections including skin abscesses, necrotizing pneumonia, joint infections, and endocarditis^1^. Methicillin-resistant *S. aureus* (MRSA) accounts for 60–70% of *S. aureus* infections in hospitals and causes the highest number of invasive infections among all antibiotic-resistant bacteria^2^. According to the U.S. Centers for Disease Control and Prevention (CDC), invasive infection of MRSA has a 14% fatality rate in 2011^3^. While the majority of MRSA cases are acquired in hospitals and other healthcare settings, community-acquired MRSA infection has seen a big increase in prevalence, posing greater danger to the public^4^,^5^,^6^. Unfortunately, new antibiotic development has not kept pace with the emergence of resistance over the past few decades^7^. Although several newer antibiotics introduced after 2000, such as linezolid and daptomycin, remain largely effective against MRSA, strains resistant to those antibiotics have already been reported^8^,^9^. Therefore, the use of antimicrobial drug combinations is increasingly seen as a critical strategy to combat multi-drug resistant pathogens such as MRSA.

Generally, drug combinations can have synergistic, additive, and antagonistic effects, depending on whether the effect of combination is bigger than, equal to or smaller than the predicted sum of the effects of individual drugs^10^. Synergistic effects are the most desirable, because lower dose can be used, which not only reduce cost and toxicity, but also slow down the development of antibiotic resistance. However, elucidation of the mechanism underlying such effects has been difficult, partly because traditional reductionist approaches mainly focused on the immediate drug targets and the addition or modification of individual cellular components that underlie the resistance^11^,^12^. This is unlikely to reveal the long chain of interactions that are likely to be responsible for synergistic effects caused by multiple drugs. Instead, systems biology approaches are more suitable to unearth the key players in the biological network which are involved^13^. In this systems view, bacteria cells respond to antibiotic damage by regulating its metabolic pathways globally to compensate for that damage^14^. Bacteria that survive the antibiotic treatment will develop persistent adaptive responses, which make it possible to develop resistance over time^15^,^16^.

Although the antimicrobial and resistance mechanisms of antibiotics have been studied for decades, much of the biology remains unknown beyond the immediate drug targets, even for the most studied pathogens. Indeed, more and more studies support the notion of a global response to antibiotic stress^17^,^18^,^19^. In a very recent study, Cho *et al*. reported that β-lactam antibiotics not only inhibit their targets, namely the penicillin binding proteins (PBPs), in *Escherichia coli* as is commonly believed, but also induce a toxic malfunctioning of the biosynthetic machinery, thereby bolstering their bactericidal activity. The cells under antibiotic stress undergo futile cycles of cell wall synthesis and degradation, which deplete cellular resources^20^. Kohanski *et al*. has demonstrated that many distinct classes of antibiotics commonly accelerate the electron transport chain via tricarboxylic acid (TCA) cycle and damage the iron-sulfur cluster, which leads to increased oxidative stress. It was thus conjectured that oxidative stress is a common antimicrobial mechanism of antibiotics^21^. Recently, proteomics has been employed as a tool to study the adaption or global response of the bacteria to the environment, including to antibiotic stress^14^,^22^,^23^,^24^. The majority of studies showed that bacterial responses to antibiotic stress are not limited to a few molecular targets directly related to the known antimicrobial and resistance mechanisms, but appear to be global. A large number of proteins involved in various pathways were differentially regulated in the presence of antibiotics. Such studies suggested that proteomics and other systems approaches to biology can potentially provide a more comprehensive picture of bacterial responses to antibiotics, complementing the traditional reductionist approach.

SIPI-8294 (Chinese patent CN201010273264 and CN201410131277) is a new derivative of erythromycin synthesized by the Shanghai Institute of Pharmaceutical Industry^25^. The chemical structures of SIPI-8294 and erythromycin are shown in Fig. 1. SIPI-8294 retains partial of the erythromycin structures, 14-membered macrolactone ring and 5-position desosamine sugar, but is more hydrophobic than erythromycin due to several different functional groups (shown in Fig. 1 with pink color). It has been revealed recently that SIPI-8294 has remarkable synergistic effect with oxacillin against MRSA *in vitro*^25^. It was shown that in the presence of a low dose of SIPI-8294, the minimum inhibitory concentration (MIC) of oxacillin was vastly reduced, as if the susceptibility of MRSA against oxacillin has been restored. Specifically, SIPI-8294 could reduce the MIC of 18 out of 21 clinically used β-lactam antibiotics against MRSA (ATCC43300) by 4 to 128 times and had synergistic effects with oxacillin against 12 out of 16 clinical isolated MRSA strains. No synergistic effects were observed against methicillin-susceptible strains. Interestingly, SIPI-8294 has no apparent bactericidal effect on MRSA, nor has its parent compound, erythromycin. Unlike SIPI-8294, however, erythromycin and other macrolides antibiotics did not exhibit any synergistic effects with the β-lactams^25^. The mechanism for the synergistic effect of SIPI-8294 and oxacillin is not known yet. This study therefore aimed at revealing the global cellular responses of MRSA against sub-MIC dose of SIPI-8294 and oxacillin, and thereby obtaining new insights into the synergistic mechanism of the drug combination.

## Results and Discussion

### Proteomic analysis

Spectral-counting based label-free quantitative proteomics was performed to investigate the synergistic effect of the combination of SIPI-8294 and oxacillin (SIPI-8294/Oxa). The workflow of sample preparation and data analysis is shown in Fig. 2. Besides the SIPI-8294/Oxa treatment group, four other treatment groups were also acquired: erythromycin/oxacillin (Ery/Oxa), oxacillin only (Oxa), SIPI-8294 only (SIPI-8294), and erythromycin only (Ery). Each treatment group was compared with the untreated control group respectively to identify differentially expressed proteins.

In all cases, sub-MIC doses of antibiotics and SIPI-8294 were applied to MRSA, so as to impose stress to the cells but not induce non-specific effects associated with cell death. In this circumstance, it is assumed that the cells will have sufficient time to change the allocation of the translational capacity to produce proteins needed to counteract the inflicted damage^15^. The growth curves for the treated cells under different conditions are shown in Supplementary Fig. S1. Under all treatment conditions including control group, MRSA continued to grow to the stationary phase and reach to the similar level after 24 hr incubation.

Figure 3 shows the total numbers of differentially expressed proteins in different treatment groups, relative to the untreated group. Figure 3a shows the volcano plot for the differentially expressed proteins in the SIPI-8294/Oxa and Ery/Oxa groups. The proteins with *p*-value of student *t*-test lower than 0.05 and fold change higher than 1.5 or lower than 0.67 (or −1.5) were considered as differentially expressed proteins. As shown in Fig. 3b, 200 proteins (120 up-regulated and 80 down-regulated) were found significantly changed in the SIPI-8294/Oxa treatment group. The number is much higher than the sum of the numbers of differentially expressed proteins in the Oxa and the SIPI-8294 treatment groups (72 and 57 differentially expressed proteins respectively). On the other hand, 124 proteins (66 up-regulated and 58 down-regulated) were differentially expressed in the Ery/Oxa treatment group, whereas 55 and 72 proteins were changed in the Ery and the Oxa treatment groups, respectively. The Venn diagram in Fig. 3c shows the number of differentially expressed proteins that are unique to the combination treatment and the individual drug treatments. As shown, 154 differentially expressed proteins were unique to the combination treatment of the SIPI-8294/Oxa treatment group, compared to 66 for the Ery/Oxa treatment group. There are 89 proteins both differentially expressed in SIPI-8294/Oxa and Ery/Oxa group. Figure 3d shows the correlation of these 89 proteins, and still a number of them couldn’t correlate well. These results indicate that the cellular response of MRSA to SIPI-8294/Oxa is different and much more complicated than Ery/Oxa, hinting at some specific mechanisms for the synergistic effect. All the differentially expressed proteins are listed in Supplementary Table S1.

**Figure 1:**
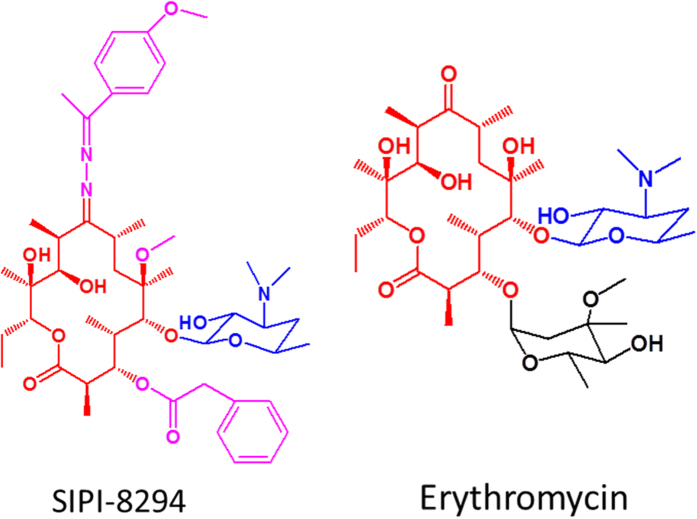
Chemical structures for SIPI-8294 and erythromycin. The macrolactone ring is in red color and the 5-position disosamine sugar is in blue color. The different functional groups between SIPI-8294 and erythromycin are in pink color for SIPI-8294 and in black color for erythromycin.

**Figure 2:**
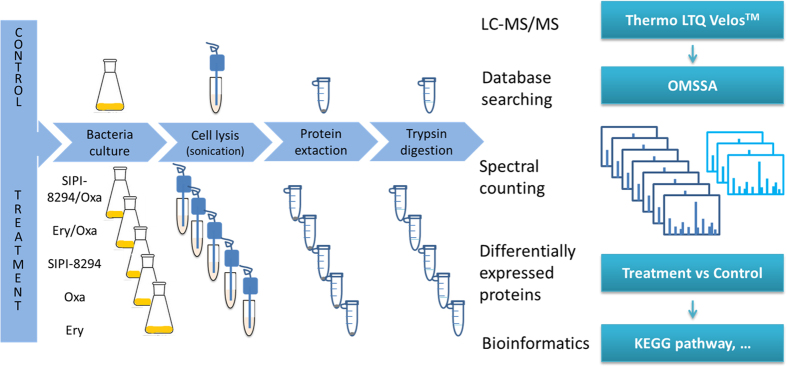
Experimental workflow for the sample preparation and data analysis. Different drug treatment groups were compared with control (no drug treatment) and performed spectral-counting based label-free quantitation.

**Figure 3:**
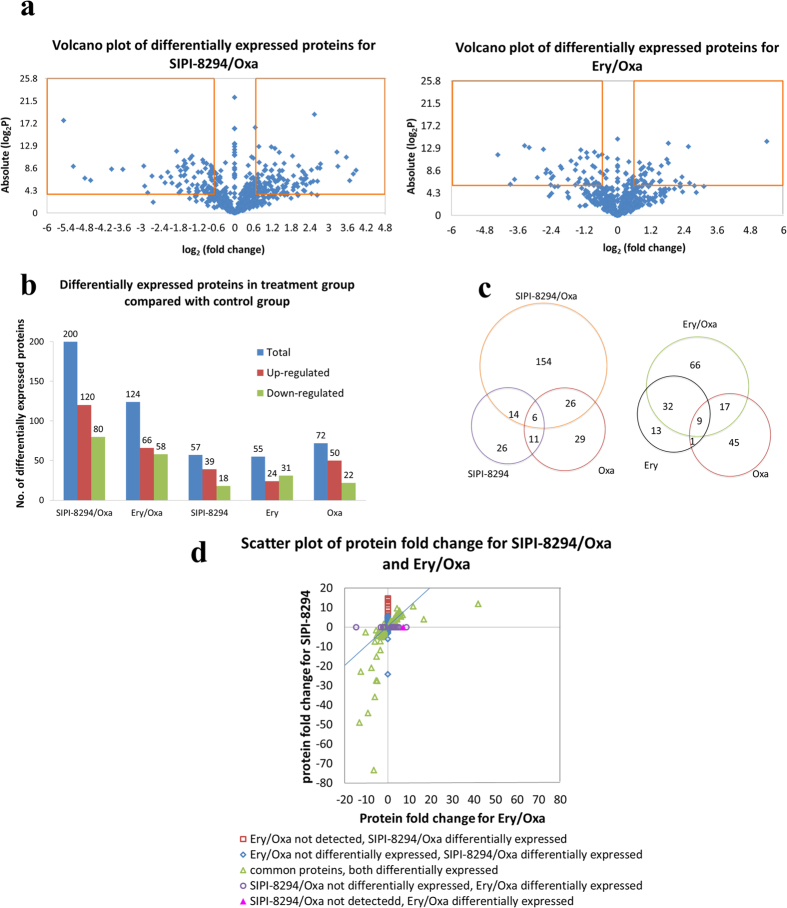
(**a**) Volcano plot of differentially expressed proteins in the SIPI-8294/Oxa and Ery/Oxa groups; (**b**) Numbers of differentially expressed proteins in different treatment groups, SIPI-8294/Oxa, Ery/Oxa, SIPI8294, Ery and Oxa, compared with control (no drug treatment); (**c**) Venn diagram for differentially expressed proteins in the SIPI-8294/Oxa and the Ery/Oxa treatment groups compared with their individual drug treatment groups; (**d**) The correlation between the differentially expressed proteins in the SIPI-8294/Oxa and Ery/Oxa groups.

**Figure 4:**
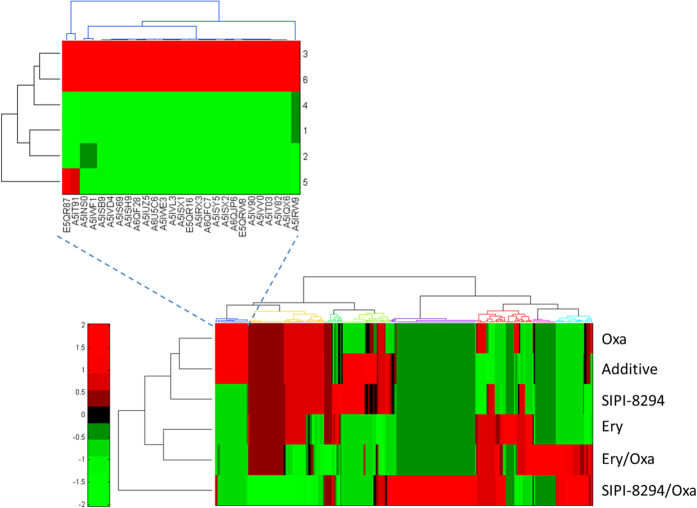
Hierarchical cluster analysis was conducted for all the differentially expressed proteins in the different treatment groups. SIPI-8294/Oxa, SIPI-8294, Oxa, Ery and Ery/Oxa, as well as the outcome that would be expected if SIPI-8294 and Oxa acted independently (Additive). Each row indicates one treatment group and each column represents one differentially expressed protein (shown in the zoom-in image). The color indicates relative fold changes (up-regulation relative to mean fold change in red, and down-regulation relative to mean fold change in green). The “Additive” group is generated by multiplying the corresponding fold changes of each differentially expressed protein in the SIPI-8294 treatment group and the Oxa treatment group.

Hierarchical cluster analysis was performed for all the differentially expressed proteins for each treatment group and a simple addition of the effects of SIPI-8294 and oxacillin (Additive) (Fig. 4). The “Additive” data-set was generated by multiplying the corresponding fold changes of each differentially expressed protein in the SIPI-8294 treatment group and the Oxa treatment group, to mimic the situation that SIPI-8294 only has simple additive effect with oxacillin. As shown in Fig. 4, the differentially expressed proteins in the SIPI-8294, Oxa and Additive treatment groups clustered together and the differentially expressed proteins in the Ery treatment group clustered with that in the Ery/Oxa treatment group, while the differentially expressed proteins in the SIPI-8294/Oxa treatment group was obviously different from all the other groups. These results suggest that SIPI-8294/Oxa elicits a cellular response that is much different from what one would expect if SIPI-8294 and Oxa act independently. Again, this provides some confidence that the observed synergistic effect does have a molecular underpinning, of which some essential elements should be captured in our data. Moreover, it is important to note that the differentially expressed proteins in the SIPI-8294 treatment group did not cluster together with that in the Ery treatment group. This suggests that the cellular responses between SIPI-8294 and erythromycin are different even though they share similar structures (Fig. 1 in red color).

To investigate the relationships between the differentially expressed proteins and the synergistic effect, all the differentially expressed proteins in the SIPI-8294/Oxa treatment group were processed by bioinformatic tools such as Megan^26^ for KEGG pathway analysis and SEED^27^ protein classification (Supplementary Fig. S2 (a) and (b)). The result shows that most assigned proteins are involved in carbohydrate metabolism (30 proteins), followed by translation (22 proteins), amino acid metabolism (17 proteins), energy metabolism (16 proteins), and nucleotide metabolism (15 proteins) etc. Moreover, all the identified proteins in the SIPI-8294/Oxa treatment group (about 1000 proteins) were also processed by the same bioinformatics tool, Megan, for KEGG pathway analysis (Supplementary Fig. S2 (c)) and SEED protein classification (Supplementary Figure S2 (d)). The KEGG pathway analyses and SEED protein classifications for all the identified proteins and the differentially expressed proteins in the SIPI-8294/Oxa treatment group exhibit different profiles. This implies that the response of the bacteria to SIPI-8294/Oxa is global and cannot be readily captured by traditional analytical techniques. To figure out what proteins are related to the synergistic effect, the subsequent analysis focuses on the proteins involved in the known oxacillin resistance mechanism, and the proteins which are changed only under SIPI-8294/Oxa treatment.

### Synergistic effect related to oxacillin resistance mechanism

Two major resistance mechanisms have been developed by bacteria to resist β-lactam antibiotics: one is the production of β-lactamase which can inactivate or degrade the β-lactam antibiotics before the antibiotics reach their targets; the other is the production of penicillin binding protein 2a (PBP2a) which has low affinity with β-lactam antibiotics to bypass the activities of antibiotics^28^,^29^. However, the β-lactam used in this work, oxacillin, cannot be hydrolyzed by β-lactamase. The main resistance mechanism to oxacillin depends on PBP2a.

**Figure 5:**
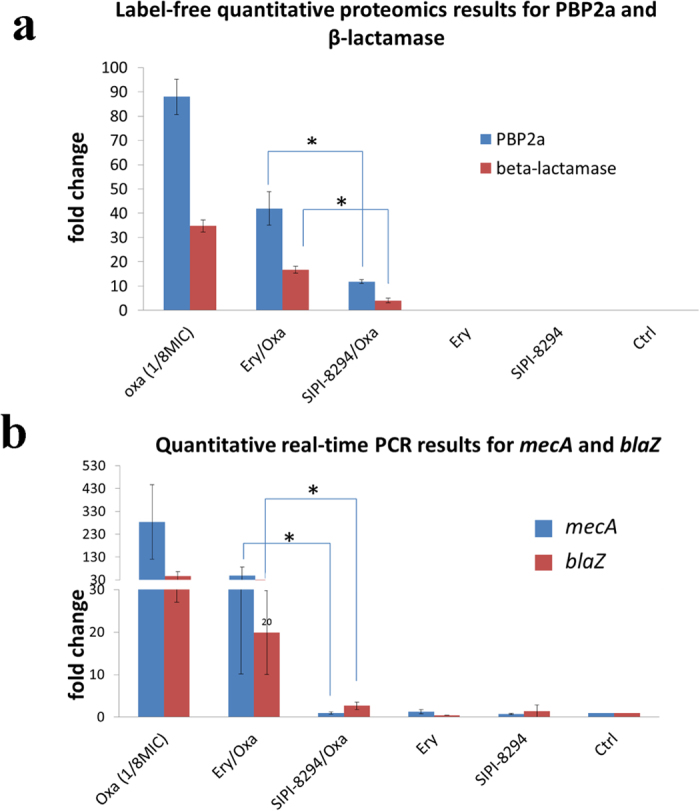
Quantification results for the expression of PBP2a (gene: *mecA*) and β-lactamase (gene*: blaZ*) at protein level by label-free quantitative proteomics method (a) and at transcription level by Q-RT-PCR method (b). PBP2a and β-lactamase are the proteins involved in resistance mechanism of bacteria against β-lactam antibiotics. Label-free quantitative proteomics results show that the levels of PBP2a and β-lactamase are lower in the SIPI-8294/Oxa than Ery/Oxa and other treatment groups. The same trend was found at the mRNA level. These results suggest that the synergistic effect mechanism may be related to the interference with the oxacillin resistance mechanism. Asterisks (*) indicate differential expression at *p* < 0.05.

As expected, PBP2a was up-regulated in all the oxacillin presented groups (the SIPI-8294/Oxa, Ery/Oxa and Oxa treatment group) and not differentially expressed in the controls, SIPI-8294 only and Ery only treatment groups. It is noted that in the SIPI-8294/Oxa and Ery/Oxa treatment groups the same doses of oxacillin were applied to the cells; however, the fold changes for PBP2a were around four times lower in the SIPI-8294/Oxa group than those in the Ery/Oxa group (Fig. 5A). Specifically, the fold changes for PBP2a were up-regulated 42.0 folds in the Ery/Oxa treatment groups whereas only up-regulated 11.8 folds in the SIPI-8294/Oxa group. These results indicate that the synergistic effect of SIPI-8294/Oxa may be due to the interference of the oxacillin resistance mechanism. Interestingly, the same phenomenon was also observed for β-lactamase even though β-lactamase should not be responsible for the resistance of oxacillin. The fold changes for β-lactamase were up-regulated 16.8 folds in the Ery/Oxa treatment group but only up-regulated 4 folds in the SIPI-8294/Oxa treatment group. To investigate whether this regulation occurs at the transcription or translation level, quantitative real-time PCR (Q-RT-PCR) was performed for both genes of PBP2a (*mecA*) and β-lactamase (*blaZ*). The results show that mRNA transcript levels of these two enzymes (*mecA* and *blaZ*) in the SIPI-8294/Oxa group were significantly lower than that in the Ery/Oxa group (*t*-test *p* < 0.05) as shown in Fig. 5b, following the same trend as the corresponding proteins. Therefore, this regulation appears to happen upstream of translation, and could not be explained by an overall reduction in protein synthesis potentially triggered by the macrolides.

### Synergistic effect related to other cellular pathways

We further focused our attention to the set of differentially expressed proteins that most strongly distinguish the SIPI-8294/Oxa and Ery/Oxa groups. Thirty-two proteins, among the 200 differentially expressed proteins in the SIPI-8294/Oxa group, exhibit a fold change over five and a ratio of (fold change of SIPI-8294/Oxa) to (fold change of Ery/Oxa) over 1.5 or lower than 0.67 as shown in Table 1. Several pathways caught our attention due to their potential roles in antibiotic responses, which we describe below.

#### Oxidative stress

**Figure 6:**
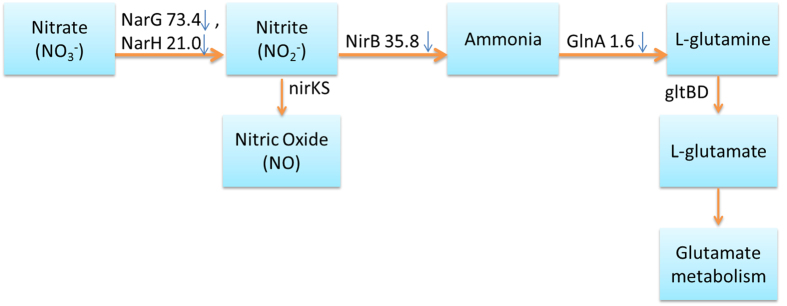
The differentially expressed proteins in the SIPI-8294/Oxa treatment group involved in nitrogen metabolism pathway classified by KEGG. *narG*: nitrate reductase alpha subunit; *narH*: respiratory nitrate reductase β subunit; *nirB*: assimilatory nitrite reductase (NAD(P)H) large subunit; NirKS: nitric oxide reductase; *glnA*: glutamine synthase; *gltBD*: glutamate synthase. The symbol of (↓) stands for down-regulated protein, and the number ahead is the fold change. *nirKS* and *gltBD* were not detected in our experiment.

Two enzymes involved in nitrogen metabolism (Fig. 6), nitrate reductase (encoded by *narG*) and respiratory nitrate reductase (*narH*), which are responsible for reducing nitrate to nitrite were dramatically down-regulated 73.4 and 21.0 folds, respectively. This may be significant since nitrite is a key source of nitric oxide, an important gasotransmitter in bacteria which has close relationship with oxidative stress and antibiotic resistance^30^,^31^. Although the level of enzyme nitric oxide reductase (encoded by *nirKS*) which reduces nitrite to nitric oxide was below the detection limit in our experiments, it is possible that the nitric oxide level will be regulated because of the down-regulation of the up-stream enzymes. It was previously reported that elimination of endogenous nitric oxide in MRSA can sensitize the cells to oxidative stress^32^. It was also conjectured that NO-mediated antibiotic resistance is achieved through both the chemical modification of toxic compounds and the alleviation of the oxidative stress imposed by many antibiotics^33^. Besides, several other important oxidoreductases were also down-regulated dramatically. Alcohol dehydrogenase (encoded by *adh*), which catalyzes the reversible reduction of acetaldehyde to ethanol, was down-regulated 49.1 folds; and L-lactate dehydrogenase 1 (encoded by *ldh1*), responsible for catalyzing lactate formation from pyruvate, was down-regulated 11.9 folds in the SIPI-8294/Oxa group. Since both alcohol dehydrogenase and L-lactate dehydrogenase 1 convert NAD^+^ to NADH in their reactions, they play important role in maintaining the redox homeostasis in bacteria^34^. Therefore, these results suggest that the oxidation-reduction homeostasis has been disturbed by the SIPI-8294/Oxa combination, which might be related to its observed synergistic effect.

#### Cell wall biosynthesis

**Figure 7:**
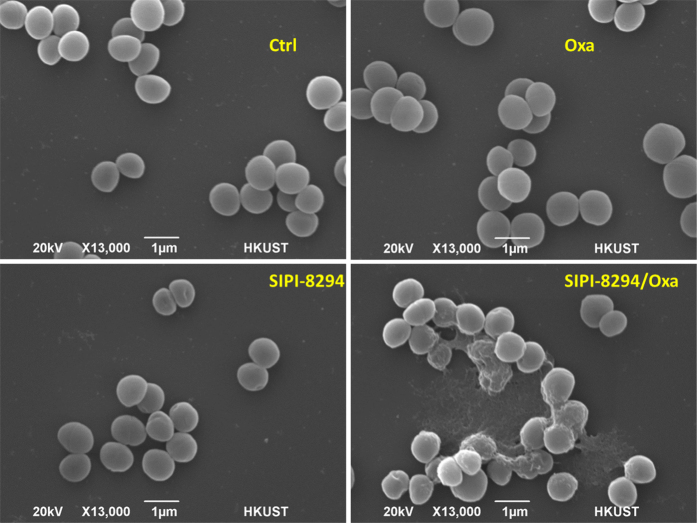
SEM images for MRSA under different drug treatments. Ctrl: Normal methicillin-resistance *S aureus* (MRSA) with no treatment; Oxa: MRSA treated with 1/8 MIC oxacillin (8 μg/ml); SIPI-8294: MRSA treated with 8 μg/ml SIPI-8294; SIPI-8294/Oxa: MRSA treated with the combination of SIPI-8294 and oxacillin (8 μg/ml for SIPI-8294 and 0.03125 μg/ml for oxacillin).

In nitrogen metabolism (Fig. 6), the enzyme assimilatory nitrite reductase (encoded by *nirB*), which reduces nitrite to ammonia, was also down-regulated by 35.8 folds. This may reduce the amount of ammonia available for synthesizing amino acids, in particular glutamine and glutamate^35^. Downstream of this pathway, glutamine synthase (encoded by *glnA*) was detected slightly down-regulated by 1.6 folds, and glutamate synthase (encoded by *gltBD*) from glutamine was below the detection limit. Glutamate and glutamine serve as the amino group donors for many nitrogen-containing compounds in at least 37 reactions^35^. Besides, glutamate is important in cell wall biosynthesis since glutamate serves as one of the amino acids in the penta-peptide component of the cell wall (Supplementary Fig. S3) and is also the monomer of poly-γ-glutamate, which is expressed on the surface of the *S. aureus* cell wall^36^. It has been reported that the inhibition of the expression and activity of glutamine synthase in *Mycobacteria* can lower the amount of poly-γ-glutamate and hamper bacteria replication^37^. Therefore, we surmise that the down-regulation of glutamate biosynthetic pathway may be detrimental to cell wall synthesis. Under the stress of the SIPI-8294/Oxa combination, it may become more difficult for the cell to maintain cell wall integrity or to produce new cell wall for replication. Since the known bactericidal effect of oxacillin is the interference with cell wall synthesis through inactivating penicillin-binding proteins (PBPs), the combination of SIPI-8294/Oxa may enhance that effect through depriving the cells of the building blocks of the cell wall. To test this hypothesis, we employed scanning electron microscope (SEM) to visualize outer surface of the cells. The SEM image for the cells in the SIPI-8294/Oxa treatment group clearly exhibit damages on the cell surface, while in other treatment conditions the cells are not visibly affected (Fig. 7).

Other differentially expressed proteins were found to involve in pathways such as glycolysis/gluconeogenesis, TCA cycle, pyruvate metabolism, pyrimidine/purine metabolism, and DNA mismatch repair (Supplementary Fig. S4). The vast number and diversity of cellular pathways affected by the SIPI-8294/Oxa treatment again confirmed that antibiotic response in bacteria is global rather than isolated, consistent with previous studies^14^,^24^.

## Conclusions

In this study, a new drug candidate SIPI-8294, derived from erythromycin and found to have synergistic effect with oxacillin against MRSA, was investigated. In order to understand the synergistic mechanism of SIPI-8294 and oxacillin, spectral counting based label-free quantitative proteomics was applied. Based on well-developed label-free quantitative proteomics workflow, sub-MIC doses of the drug combination of SIPI-8294/Oxa and Ery/Oxa as well as SIPI-8294 only, oxacillin only and erythromycin only were applied to MRSA. The differentially expressed proteins were obtained by comparing the drug treatment groups with the untreated control group. Hierarchical clustering analysis shows that SIPI-8294/Oxa elicits very different responses in the cell than those by the individual drugs or the Ery/Oxa combination, which shows no synergistic effect.

Moreover, the differentially expression levels of PBP2a was four times lower in the SIPI-8294/Oxa group than those in the Ery/Oxa group in label-free quantitative proteomics results. In addition, mRNA transcription levels also have the same trend. These results indicate that the synergistic mechanism may be related to the interference with oxacillin resistance mechanism. We also observed large fold changes in proteins spanning many different pathways, indicating a global response of the bacteria to the antibiotics. Although we cannot distinguish between cause (the action of the antibiotic) and effect (the response of the bacteria to combat such action) using our data, we identified oxidation-reduction homeostasis and cell wall biosynthesis to be possible players in mechanism of the synergistic effect of SIPI-8294 and oxacillin. Since the cells were still growing under our conditions, it would be expected that the cells can counteract the stress imposed on them sufficiently to restore the homeostatic balance required for continued growth. However, our data would reveal which aspects of the cellular machinery have been perturbed, from which potential hypotheses about cause and effect can be formulated.

## Methods

### Minimum inhibitory concentration (MIC) tests

The *S. aureus* strains ATCC 43300 (MRSA) was obtained from the American Type Culture Collection. The bacteria were cultured in cation-adjusted Mueller-Hinton broth at 35 ± 2 °C. Minimum inhibitory concentration (MIC) test was performed according to the Clinical and Laboratory Standards Institute (CLSI) standard to verify the antibiotic effect. The MIC was determined to be the lowest concentration at which no visible growth of bacteria can be observed after incubation for 24 hours.

The synergistic effect was evaluated by the fractional inhibitory concentration (FIC) index based on the Loewe additivity zero-interaction theory^38^. For the combination A and B, it was calculated as FIC = MIC_(A in combination)_/MIC_(A alone)_ + MIC_(B in combination)_/MIC_(B alone)_. When FIC index is lower than or equal to 0.5, the combination is considered to have a synergistic effect. The measured MIC and FIC values were given in Supplementary Table S2.

### Cell culture and drug treatment

The bacteria were grown in cation-adjusted Mueller-Hinton broth (Sigma-Aldrich, St. Louis, MO, USA). The bacteria without dosing any antibiotics were considered as untreated control. For antibiotic treatment, 1/8 MIC of antibiotics were added to 100 mL of medium and cultured together with 10^6^/mL seeded bacteria. For the combination of SIPI-8294 and oxicillin (SIPI-8294/Oxa) treatment group, 8 μg/mL of SIPI-8294 was used in this work because the lowest FIC index was obtained at this concentration (as shown in Supplementary Table S2). The concentration for oxacillin in the SIPI-8294/Oxa group was taken at 1/8 MIC of oxacillin in the presence of 8 μg/mL SIPI-8294, which is 0.03125 μg/mL. For the combination of erythromycin and oxacillin (Ery/Oxa) treatment group, the concentrations used in this work were 32 μg/mL and 0.03125 μg/mL for erythromycin and oxacillin, respectively. For the single drug treatment, SIPI-8294 was added at the concentration of 8 μg/mL; oxacllin was added at the concentration of 8 μg/mL; erythromycin was added at the concentration of 32 μg/mL.

Three biological replicates were performed for each condition and two technical replicates were performed for each biological replicate. Bacteria growth was measured by OD_600_ (optical density at 600 nm). The cells were harvested when OD_600_ reached 0.1.

### Label-free quantitative proteomics

The cells were collected and washed with phosphate buffered saline (PBS) buffer twice. Cell disruption was completed by ultra-sonication in the lysis buffer (8 M urea) for 5 min under ice-water bath. Then the cell lysate was centrifuged at 4000 rpm for 10 min at 4 °C and the supernatant was subjected to cold acetone precipitation. The precipitated proteins were dissolved in 4 M urea and 30 mM Tris-HCl (pH 6.5) and the protein concentration was measured by Bradford Protein assay (Bio-Rad, Hercules, CA, U. S. A.).

Thirty micrograms of proteins were used for the proteomics sample preparation, which was reduced by dithiothreitol (DTT) and alkylated by iodoacetamide. The resulting proteins were then digested by trypsin (1: 50 w/w, Promega, Madsion, WI) overnight at 37 °C. After digestion, the peptide sample was desalted by C18 reverse-phase ZipTip (Millipore, Darmstadt, Germany) and dried by SpeedVac (Eppendorf, Hamburg, Germany). Unless otherwise noted, all reagents were purchased from Sigma-Aldrich (St. Louis, MO, U.S.A).

The peptide samples were analyzed on a Thermo Scientific LTQ VelosTM platform (Thermo Fisher Scientific, Bremen, Germany) coupled with a Thermo Accela LC. One microgram of peptides were enriched on a trap column (Zorbax X300 SB-C18, 5 × 0.3 mm, 5 μm particle size) and separated on a C18 column (Thermo Bio-Basic-18, 150 × 0.1 mm, 300 Å pore size, 5 μm particle size) at a flow rate of 150 μL/min and 150 min LC run. For the MS parameters, the top ten most intense ions observed in the MS1 scan were set to acquire MS2 spectra. The dynamics exclusion was set as 60 seconds and the normalized collision energy was set at 30%.

MM file conversion (v3.9)^39^ was utilized to convert all the raw data into mgf file, and OMSSA^40^ was applied to search all the files against MRSA database. The database was constructed by combining complete proteome of 23 strains MRSA in UniProt, 1:1 ratio shuffled decoy protein sequences and common contaminants. Carbamidomethylation on cysteine and oxidation on methionine were set as fix modification and variable modification, respectively. The search results were further processed by the Trans-Proteomic Pipeline (TPP)^41^. The spectral counts of the two technical replicates from the same biological replicate with protein FDR lower than 0.01 were combined.

For the label-free comparative statistical analysis, the proteins identified confidently in at least two out of three biological replicates and the average spectral counts equal or over five were included in the statistical analysis. The spectral count of each protein was normalized by the total spectral counts of the biological replicate^42^,^43^. The statistical analysis (Student’s *t*-test and G-test) was conducted by PepC^44^ on every two conditions: antibiotic treated versus untreated control group. The differentially expressed proteins were filtered by the following cutoff: *p*-value (*t*-test) was lower than 0.05; and the fold changes were higher than 1.5 fold.

### Bioinformatic analysis

Protein hierarchical analysis was done by Matlab. Protein localization prediction was done by the automatic bioinformatic pipeline named SLEP (surface localization of extracellular proteins)^45^ and PSORTb v3.0^46^. Differentially expressed proteins were blasted against the NCBI-NR database of non-redundant protein sequences, and input into MEGAN^47^ to perform functional analysis using the SEED classification^48^ of subsystems and functional roles or the KEGG classification of pathways and enzymes.

### Q-RT-PCR analysis of *mecA* and *blaZ* gene expression

The expression of two oxacillin resistance-related genes (*mecA*, coding for PBP2a, and *blaZ*, coding for β-lactamase) was quantified by real-time PCR. MRSA cells were cultured in the same condition as the proteomics experiments with five drug treatment groups and one untreated control group. In order to stabilize RNA, RNALater® (Ambion, Austin, TX) was added to the cells immediately after the cells being collected. Total RNA was extracted using AllPrep DNA/RNA Mini Kit (Qiagen, California, U.S.A) according to the manufacturer’s instruction. Q-RT-PCR was performed by a two-step process. RNA was first reverse transcribed to cDNA (Invitrogen, Carlsbad, CA); and RT-PCR was conducted on 7500 Fast RT-PCR (Applied Biosystems, California, U.S.A) using KAPA SYBR® FAST qPCR Kit with 40 cycles of denaturation for 5 seconds at 95 °C, annealing for 30 seconds at 50 °C, and extension for 20 seconds at 72 °C PCR primers for the *mecA* gene were (F: GTTAGATTGGGATCATAGCGTCATT) and (R: GCCTAATCTCATATGTGTTC CTGTAT); for *blaZ* gene were (F: CGTCTAAAAGAACTAGGAG) and (R: GCTTAA TTTTCCATTTGCGATAAG) and for 16S rRNA were (F: TCCGGAATTATTGGGCGTAA) and (R: CCACTTTCCTCTTCTGCACTCA). The melting curve analysis was performed immediately after amplification to verify the specificity of the PCR amplification products.

Fluorescence was measured at the end of the annealing-extension phase of each cycle. A threshold value for the fluorescence of all samples was set manually. The reaction cycle at which the PCR product exceeds this fluorescence threshold was identified as the threshold cycle (CT). The relative quantitation was calculated by the 2^−ΔΔCT^ method^49^.

### SEM

The cells were grown in SIPI-8294/Oxa (8/0.03125 μg/mL), SIPI-8294 (8 μg/mL), Oxacillin (8 μg/mL) and untreated control medium to OD 0.1. The cells were fixed in 2.5% glutaraldehyde (PBS buffer) for 2 hours, and washed with PBS buffer twice after fixation. Then the cells were dehydration in an ethanol series with increasing concentration (30%, 40%, 50%, 60%, 70%, 80%, 90%, pure ethanol) for 15 min, and finally suspend in pure tert-butanol for 15 min. The cells were air-dried and coated with gold followed by scanning electron microscope analysis (JSM-6390 Scanning Electron Microscope).

## Additional Information

**How to cite this article**: Liu, X. *et al*. Proteomic response of methicillin-resistant *S. aureus* to a synergistic antibacterial drug combination: a novel erythromycin derivative and oxacillin. *Sci. Rep.* **6**, 19841; doi: 10.1038/srep19841 (2016).

## Supplementary Material

Supplementary Information

## Figures and Tables

**Table 1:** Differentially expressed proteins potentially related to synergistic mechanism.

Uniprot_AC	Gene name	Protein name	Fold changes		Cellular location	Pathway
			8294+Oxa	Ery+Oxa		
A6QJN7	narG	Nitrate reductase, alpha subunit	(−)73.4 (Repression)	(−)6.4	Membrane	Nitrogen metabolism
A5IQF9	adh	Alcohol dehydrogenase	(−)49.1 (Repression)	(−)13.1	CytopPlasmic	Tyrosine metabolism; Fatty acid metabolism; Glycolysis/Gluconeogenesis;
E5QWJ9	HMPREF0772_10696	LPXTG-motif cell wall anchor domain protein	(−)44.1	(−)9.1	Cell wall	
A5IVI0	nirB	Assimilatory nitrite reductase (NAD(P)H) large subunit (EC 1.7.1.4)	(−)35.8	(−)6.0	Cytoplasmic	
Q6GGX3	ebh SAR1447	Extracellular matrix-binding protein ebh (ECM-binding protein homolog)	(−)27.7 (Repression)	(−)4.8	Cell wall	
A8Z0I5	pfl	Formate C-acetyltransferase (EC 2.3.1.54)	(−)27.5	(−)5.3	Cytoplasmic	Butanoate metabolism; Propanoate metabolism; Pyruvate metabolism
E5QTK7	sdrD	Serine-aspartate repeat-containing protein D	(−)24.3	—	Cell wall	
A5IUP1	sceD SaurJH9_2132	Transglycosylase domain protein, probable transglycosylase SceD	(−)22.9 (Repression)	(−)12.5 (Repression)	Extracellular	
A5IVH7	narH	Respiratory nitrate reductase β subunit	(−)21 (Repression)	(−)7.5	CytoplasmicMembrane	Nitrogen metabolism
A5ISU2	SaurJH9_1471	Uncharacterized protein	(−)15.2	(−)5.2	Unknown	
A5IPA9	ldh1	L-lactate dehydrogenase 1 (L-LDH 1) (EC 1.1.1.27)	(−)11.9	(−)3.5	Cytoplasmic	Cysteine and methionine metabolism; Glycolysis/Gluconeogenesis; Propanoate metabolism; Pyruvate metabolism
E5QSI9	sle	N-acetylmuramoyl-L-alanine amidase Sle1 (EC 3.5.1.28)	(−)7.3	(−)3.5	Cell wall	
A5IVG3	SaurJH9_2406	Pyridoxamine 5'-phosphate oxidase-related, FMN-binding	(−)6.2	—	Unknown	
A5IQZ6	SaurJH9_0817	Cold-shock DNA-binding protein family	(−)5.1	(−)2.9	Cytoplasmic	
A5IWF1	blaZ	β-Lactamase (EC 3.5.2.6)	(+)4 (Induction)	(+)16.8 (Induction)	Cell wall	β-Lactam resistance
E5QS79	uvrB	UvrABC system protein B (Protein UvrB) (Excinuclease ABC subunit B)	(+)5.3 (Induction)	ND	Cytoplasmic	Nucleotide excision repair
A5IUI6	SaurJH9_2077	PfkB domain protein	(+)5.4	ND	Cytoplasmic	Amino sugar and nucleotide sugar metabolism; Fructose and mannose metabolism; Starch and sucrose metabolism
A5IW34	SaurJH9_2631	AMP-dependent synthetase and ligase	(+)5.5	—	Cytoplasmic	Carbon fixation pathways in prokaryotes; Glycolysis/Gluconeogenesis; Propanoate metabolism; Pyruvate metabolism
A5IST2	lysA	Diaminopimelate decarboxylase (DAP decarboxylase) (DAPDC) (EC 4.1.1.20)	(+)5.5 (Induction)	—	Cytoplasmic	Lysine biosynthesis
A5ISH1	SaurJH9_1347	Uncharacterized protein	(+)5.7	—	Cytoplasmic	
A5ISS3	SaurJH9_1452	Uncharacterized protein-like protein	(+)6.1 (Induction)	(+)3.8 (Induction)	Cytoplasmic	
A6QFW9	pycA	Pyruvate carboxylase (EC 6.4.1.1)	(+)6.7	ND	Cytoplasmic	Citrate cycle (TCA cycle); Carbon fixation pathways in prokaryotes; Pyruvate metabolism
E5QS29	nadE	NH(3)-dependent NAD(+) synthetase (EC 6.3.1.5)	(+)8.4	(+)5.5 (Induction)	Cytoplasmic	Nicotinate and nicotinamide metabolism
A5ISV5	msrA	Peptide methionine sulfoxide reductase MsrA (EC 1.8.4.11)	(+)8.5	—	Extracellular	
A6QI23	prsA	Foldase protein PrsA (EC 5.2.1.8)	(+)9.6	(+)4.3	CytoplasmicMembrane	
A8Z1L4	purH	Bifunctional purine biosynthesis protein PurH	(+)9.8	ND	Cytoplasmic	One carbon pool by folate; Purine metabolism
A5INS0	mecA	Penicillin binding protein 2a (EC 2.4.1.129)	(+)11.8	(+)42.0	CytoplasmicMembrane	β-Lactam resistance
A5IU99	SaurJH9_1988	ABC transporter related	(+)12.2 (Induction)	ND	CytoplasmicMembrane	ABC transporters
A5ISH6	miaB	(Dimethylallyl)adenosine tRNA methylthiotransferase MiaB (EC 2.-.-.-)	(+)12.8	ND	Cytoplasmic	
Q6GEW4	mnaA	UDP-N-acetylglucosamine 2-epimerase (EC 5.1.3.14)	(+)13.8	ND	Cytoplasmic	Amino sugar and nucleotide sugar metabolism
A5IV74	ureG	Urease accessory protein UreG	(+)13.9 (Induction)	ND	Cytoplasmic	
A5INQ7	SaurJH9_0016	Primary replicative DNA helicase (EC 3.6.1.-)	(+)14.7	ND	Cytoplasmic	DNA replication; Alanine, aspartate and glutamate metabolism; Purine metabolism

Cutoff: Fold change _(SIPI-8294/Oxa)_ >5; Fold change _(SIPI-8294/Oxa)_ /Fold change _(Ery/Oxa)_ >1.5 or <0.67. Note: The fold changes with (+) stand for up-regulated proteins and those with (−) stand for down-regulated proteins. The symbol of “−” stands for non-differentially expressed protein and “ND” stands for non-detected protein. The proteins which cannot be detected in the blank but detected in the drug treatment group are considered as “induction”; the proteins which can be detected in the blank but cannot be detected in the drug treatment group are considered as “repression”. For the induction or repression proteins, the spectral counts are set to 1 for non-detected proteins to calculate fold changes.

## References

[b1] Kluytmans J., Van Belkum A. & Verbrugh H. Nasal carriage of Staphylococcus aureus: epidemiology, underlying mechanisms, and associated risks. Clin Microbiol Rev 10, 505–520 (1997). 9227864 10.1128/cmr.10.3.505 PMC172932

[b2] Taubes G. The bacteria fight back. Science (New York, NY) 321, 356 (2008). 10.1126/science.321.5887.356 18635788

[b3] Klevens R. M. . Invasive methicillin-resistant staphylococcus aureus infections in the united states. JAMA 298, 1763–1771 (2007). 17940231 10.1001/jama.298.15.1763

[b4] Morens D. M. & Fauci A. S. Emerging Infectious Diseases: Threats to Human Health and Global Stability. PLoS Pathogens 9, e1003467 (2013). 23853589 10.1371/journal.ppat.1003467 PMC3701702

[b5] DeLeo F. R. & Chambers H. F. Reemergence of antibiotic-resistant Staphylococcus aureus in the genomics era. J. Clin Invest 119, 2464–2474 (2009). 19729844 10.1172/JCI38226 PMC2735934

[b6] Chambers H. F. The changing epidemiology of Staphylococcus aureus? Emerg Infect Dis 7, 178 (2001). 11294701 10.3201/eid0702.010204 PMC2631711

[b7] Williams A. J. . Open PHACTS: semantic interoperability for drug discovery. Drug Discov Today 17, 1188–1198 (2012). 22683805 10.1016/j.drudis.2012.05.016

[b8] Mangili A., Bica I., Snydman D. & Hamer D. Daptomycin-resistant, methicillin-resistant Staphylococcus aureus bacteremia. Clin Infect Dis 40, 1058–1060 (2005). 15825002 10.1086/428616

[b9] Tsiodras S. . Linezolid resistance in a clinical isolate of *Staphylococcus aureus*. Lancet 358, 207–208 (2001). 11476839 10.1016/S0140-6736(01)05410-1

[b10] Chait R., Craney A. & Kishony R. Antibiotic interactions that select against resistance. Nature 446, 668–671 (2007). 17410176 10.1038/nature05685

[b11] Alekshun M. N. & Levy S. B. Molecular mechanisms of antibacterial multidrug resistance. Cell 128, 1037–1050 (2007). 17382878 10.1016/j.cell.2007.03.004

[b12] Berger-Bachi B. Resistance mechanisms of gram-positive bacteria. Int J Med Microbiol 292, 27–35 (2002). 12139425 10.1078/1438-4221-00185

[b13] Jia J. . Mechanisms of drug combinations: interaction and network perspectives. *Nature reviews*. Drug Discov 8, 111–128 (2009). 10.1038/nrd2683 19180105

[b14] Lima T. B. . Bacterial resistance mechanism: what proteomics can elucidate. FASEB J 27, 1291–1303 (2013). 23349550 10.1096/fj.12-221127

[b15] Wenzel M. & Bandow J. E. Proteomic signatures in antibiotic research. Proteomics 11, 3256–3268 (2011). 21726050 10.1002/pmic.201100046

[b16] Burchmore R. Mapping pathways to drug resistance with proteomics. Expert Rev Proteomics 11, 1–3 (2014). 24351106 10.1586/14789450.2014.871497

[b17] Shatalin K., Shatalina E., Mironov A. & Nudler E. H2S: a universal defense against antibiotics in bacteria. Science 334, 986–990 (2011). 22096201 10.1126/science.1209855

[b18] Fajardo A. & Martinez J. L. Antibiotics as signals that trigger specific bacterial responses. Curr Opin Microbiol 11, 161–167 (2008). 18373943 10.1016/j.mib.2008.02.006

[b19] Belenky P. & Collins J. J. Antioxidant strategies to tolerate antibiotics. Sci Signal 334, 915 (2011). 10.1126/science.1214823 22096180

[b20] Cho H., Uehara T. & Bernhardt T. G. Beta-Lactam Antibiotics Induce a Lethal Malfunctioning of the Bacterial Cell Wall Synthesis Machinery. Cell 159, 1300–1311 (2014). 25480295 10.1016/j.cell.2014.11.017 PMC4258230

[b21] Kohanski M. A., Dwyer D. J., Hayete B., Lawrence C. A. & Collins J. J. A common mechanism of cellular death induced by bactericidal antibiotics. Cell 130, 797–810 (2007). 17803904 10.1016/j.cell.2007.06.049

[b22] Van Oudenhove L. & Devreese B. A review on recent developments in mass spectrometry instrumentation and quantitative tools advancing bacterial proteomics. Appl Microbiol Biotechnol 97, 4749–4762 (2013). 23624659 10.1007/s00253-013-4897-7

[b23] Robinson J. L., Adolfsen K. J. & Brynildsen M. P. Deciphering nitric oxide stress in bacteria with quantitative modeling. Curr Opin Microbiol 19, 16–24 (2014). 24983704 10.1016/j.mib.2014.05.018 PMC4130159

[b24] Vranakis I. . Proteome studies of bacterial antibiotic resistance mechanisms. J Proteomics 97, 88–99 (2014). 24184230 10.1016/j.jprot.2013.10.027

[b25] Zhonglei L. . New erythromycin derivatives can enhance beta-lactam antibiotics against Methicillin-resistant *Staphylococcus aureus*. Lett Appl Microbiol 60 (4), 352–358 (2015). 25588530 10.1111/lam.12378

[b26] Huson D. H., Mitra S., Ruscheweyh H. J., Weber N. & Schuster S. C. Integrative analysis of environmental sequences using MEGAN4. Genome Res 21, 1552–1560, (2011). 21690186 10.1101/gr.120618.111 PMC3166839

[b27] Overbeek R. . The subsystems approach to genome annotation and its use in the project to annotate 1000 genomes. Nucleic Acids Res 33, 5691–5702 (2005). 16214803 10.1093/nar/gki866 PMC1251668

[b28] Poole K. Resistance to β-lactam antibiotics. Cell Mol Life Sciences CMLS 61, 2200–2223 (2004). 10.1007/s00018-004-4060-9 PMC11138534 15338052

[b29] Davies J. & Davies D. Origins and evolution of antibiotic resistance. Microbiol Mol Biol Rev: MMBR 74, 417–433 (2010). 20805405 10.1128/MMBR.00016-10 PMC2937522

[b30] Zumft W. G. Cell biology and molecular basis of denitrification. Microbiol Mol Biol Rev 61, 533–616 (1997). 9409151 10.1128/mmbr.61.4.533-616.1997 PMC232623

[b31] Crane B. R., Sudhamsu J. & Patel B. A. Bacterial nitric oxide synthases. Annu Rev Biochem 79, 445–470 (2010). 20370423 10.1146/annurev-biochem-062608-103436

[b32] Van Sorge N. M. . Methicillin-resistant *Staphylococcus aureus* Bacterial Nitric-oxide Synthase Affects Antibiotic Sensitivity and Skin Abscess Development. J Biolog Chem 288, 6417–6426 (2013). 10.1074/jbc.M112.448738 PMC3585076 23322784

[b33] Gusarov I., Shatalin K., Starodubtseva M. & Nudler E. Endogenous nitric oxide protects bacteria against a wide spectrum of antibiotics. Science 325, 1380–1384, (2009). 19745150 10.1126/science.1175439 PMC2929644

[b34] Richardson A. R., Libby S. J. & Fang F. C. A Nitric Oxide–Inducible Lactate Dehydrogenase Enables Staphylococcus aureus to Resist Innate Immunity. Science 319, 1672–1676 (2008). 18356528 10.1126/science.1155207

[b35] Gunka K. & Commichau F. M. Control of glutamate homeostasis in Bacillus subtilis: a complex interplay between ammonium assimilation, glutamate biosynthesis and degradation. Mol Microbiol 85, 213–224 (2012). 22625175 10.1111/j.1365-2958.2012.08105.x

[b36] Candela T. & Fouet A. Poly-gamma-glutamate in bacteria. Mol Microbiol 60, 1091–1098 (2006). 16689787 10.1111/j.1365-2958.2006.05179.x

[b37] Harth G., Zamecnik P. C., Tang J.-Y., Tabatadze D. & Horwitz M. A. Treatment of Mycobacterium tuberculosis with antisense oligonucleotides to glutamine synthetase mRNA inhibits glutamine synthetase activity, formation of the poly-L-glutamate/glutamine cell wall structure, and bacterial replication. Proc Nat Acad Sci 97, 418–423 (2000). 10618433 10.1073/pnas.97.1.418 PMC26678

[b38] Loewe S. The problem of synergism and antagonism of combined drugs. Arzneimittelforschung 3, 285–290 (1953). 13081480

[b39] Xu H. & Freitas M. A. Monte Carlo simulation-based algorithms for analysis of shotgun proteomic data. J Proteome Res 7, 2605–2615 (2008). 18543962 10.1021/pr800002u PMC2749500

[b40] Geer L. Y. . Open mass spectrometry search algorithm. J Proteome Res 3, 958–964 (2004). 15473683 10.1021/pr0499491

[b41] Deutsch E. W. . A guided tour of the Trans-Proteomic Pipeline. Proteomics 10, 1150–1159 (2010). 20101611 10.1002/pmic.200900375 PMC3017125

[b42] Carvalho P. C., Fischer J. S., Chen E. I., Yates J. R. & Barbosa V. C. PatternLab for proteomics: a tool for differential shotgun proteomics. BMC Bioinformatics 9 (2008). 10.1186/1471-2105-9-316 PMC2488363 18644148

[b43] Carvalho P. C., Hewel J., Barbosa V. C. & Yates J. R. Identifying differences in protein expression levels by spectral counting and feature selection. Genet Mol Res 7, 342–356 (2008). 18551400 10.4238/vol7-2gmr426 PMC2703009

[b44] Heinecke N., Pratt B., Vaisar T. & Becker L. PepC: proteomics software for identifying differentially expressed proteins based on spectral counting. Bioinformatics 26, 1574–1575 (2010). 20413636 10.1093/bioinformatics/btq171 PMC2881356

[b45] Giombini E., Orsini M., Carrabino D. & Tramontano A. An automatic method for identifying surface proteins in bacteria: SLEP. BMC bioinformatics 11, 39 (2010). 20089159 10.1186/1471-2105-11-39 PMC2832898

[b46] Yu N. Y. . PSORTb 3.0: improved protein subcellular localization prediction with refined localization subcategories and predictive capabilities for all prokaryotes. Bioinformatics 26, 1608–1615 (2010). 20472543 10.1093/bioinformatics/btq249 PMC2887053

[b47] Huson D. H., Mitra S., Ruscheweyh H.-J., Weber N. & Schuster S. C. Integrative analysis of environmental sequences using MEGAN4. Genome Res 21, 1552–1560 (2011). 21690186 10.1101/gr.120618.111 PMC3166839

[b48] Overbeek R. . The subsystems approach to genome annotation and its use in the project to annotate 1000 genomes. Nucleic Acids Res 33, 5691–5702 (2005). 16214803 10.1093/nar/gki866 PMC1251668

[b49] Schmittgen T. D. & Livak K. J. Analyzing real-time PCR data by the comparative CT method. Nat Protoc 3, 1101–1108 (2008). 18546601 10.1038/nprot.2008.73

