# Supplementary material for: Proteomic response of methicillin-resistant S. aureus to a synergistic antibacterial drug combination: a novel erythromycin derivative and oxacillin
Source: Sci Rep. 2016 Jan 25;6:19841. doi: 10.1038/srep19841 (PMC4726183; doi:10.1038/srep19841)
Supplement: Supplementary Information [file srep19841-s1.pdf]

Supporting Information

Proteomic response of methicillin-resistant *S. aureus* to a synergistic antibacterial drug combination: a novel erythromycin derivative and oxacillin

Xiaofen Liu [1,2,3§], Pei-Jin Pai [1,4,5§], Weipeng Zhang [6], Yingwei Hu [1], Xiaojing Dong [7], Pei-yuan Qian [6], Daijie Chen [7,8*], Henry Lam [1,9*]

1 Department of Chemical and Biomolecular Engineering, The Hong Kong University of Science and Technology, Clear Water Bay, Kowloon, Hong Kong

2 Institute of Antibiotics, Huashan Hospital affiliated to Fudan University, Shanghai, China

3 Key Laboratory of Clinical Pharmacology of Antibiotics, National Population and Family Planning Commission, Shanghai, China

4 Graduate School of Medicine, Kaohsiung Medical University, Kaohsiung, Taiwan

5 Center for Infectious Disease and Cancer Research, Kaohsiung Medical University, Kaohsiung, Taiwan

6 KAUST Global Partnership program, Division of Life Science, The Hong Kong University of Science and Technology, Clear Water Bay, Kowloon, Hong Kong

7 State Key Laboratory of New Drug and Pharmaceutical Process, Shanghai Institute of Pharmaceutical Industry, China State Institute of Pharmaceutical Industry, Shanghai, China

8 School of Pharmacy, Shanghai Jiao Tong University, Shanghai, China

9 Division of Biomedical Engineering, The Hong Kong University of Science and Technology, Clear Water Bay, Kowloon, Hong Kong

[§] Xiaofen Liu and Pei-Jing Pai contributed equally to this work.

* Correspondence should be addressed to H. N. Lam (kehlam@ust.hk), D. Chen (hccb001@163.com).

Table of contents

Supplementary Figures

Figure S1: Growth Curves for the experimental conditions.

Figure S2: KEGG pathway (a) and SEED analysis (b) for the differentially expressed proteins in SIPI-8294/Oxa treatment group; KEGG pathway (c) and SEED analysis (d) for all the identified proteins in SIPI-8294/Oxa treatment group

Figure S3: The enzymes involved in cell wall biosynthesis.

Figure S4: (a) The enzymes differentially expressed in carbohydrate metabolism pathways; * stands for AMP-dependent synthetase and ligase; (b) the enzymes differentially expressed in nitrogen and purine/pyrimidine metabolism pathways.

Supplementary Tables

Table S1: The differentially expressed proteins in different treatment groups

Table S2. Minimum inhibitory concentrations (MIC) of oxacillin in the presence of SIPI-8294 and erythromycin against MRSA (ATCC43300), and the fractional inhibitory concentration (FIC) indexes in each case.

Supplementary Figures

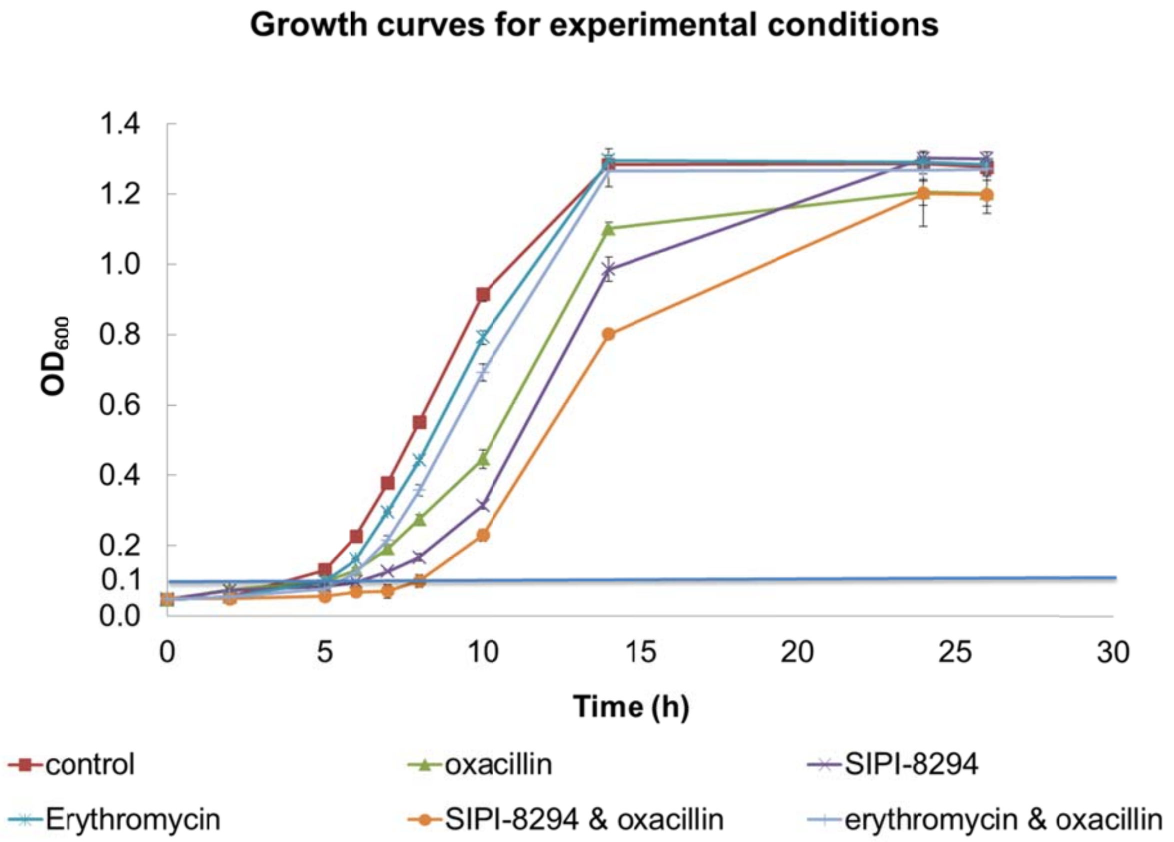

Figure S1: Growth Curves for the experimental conditions. Control: MRSA cells without drug treatment; Oxacillin: MRSA cells treated with 1/8 MIC of oxacillin (8 μg/mL); SIPI-8294: MRSA cells treated with 8 μg/mL SIPI-8294; Erythromycin: MRSA cells treated with 1/8 MIC of erythromycin (32 μg/mL); SIPI-8294/Oxa: MRSA cells treated with 8 μg/mL SIPI-8294 and 0.03125 μg/mL oxacillin; Ery/Oxa: MRSA cells treated with 32 μg/mL erythromycin and 0.03125 μg/mL oxacillin.In the proteomic analysis, all the cells were collected when OD_{600} reached 0.1 (the blue line).

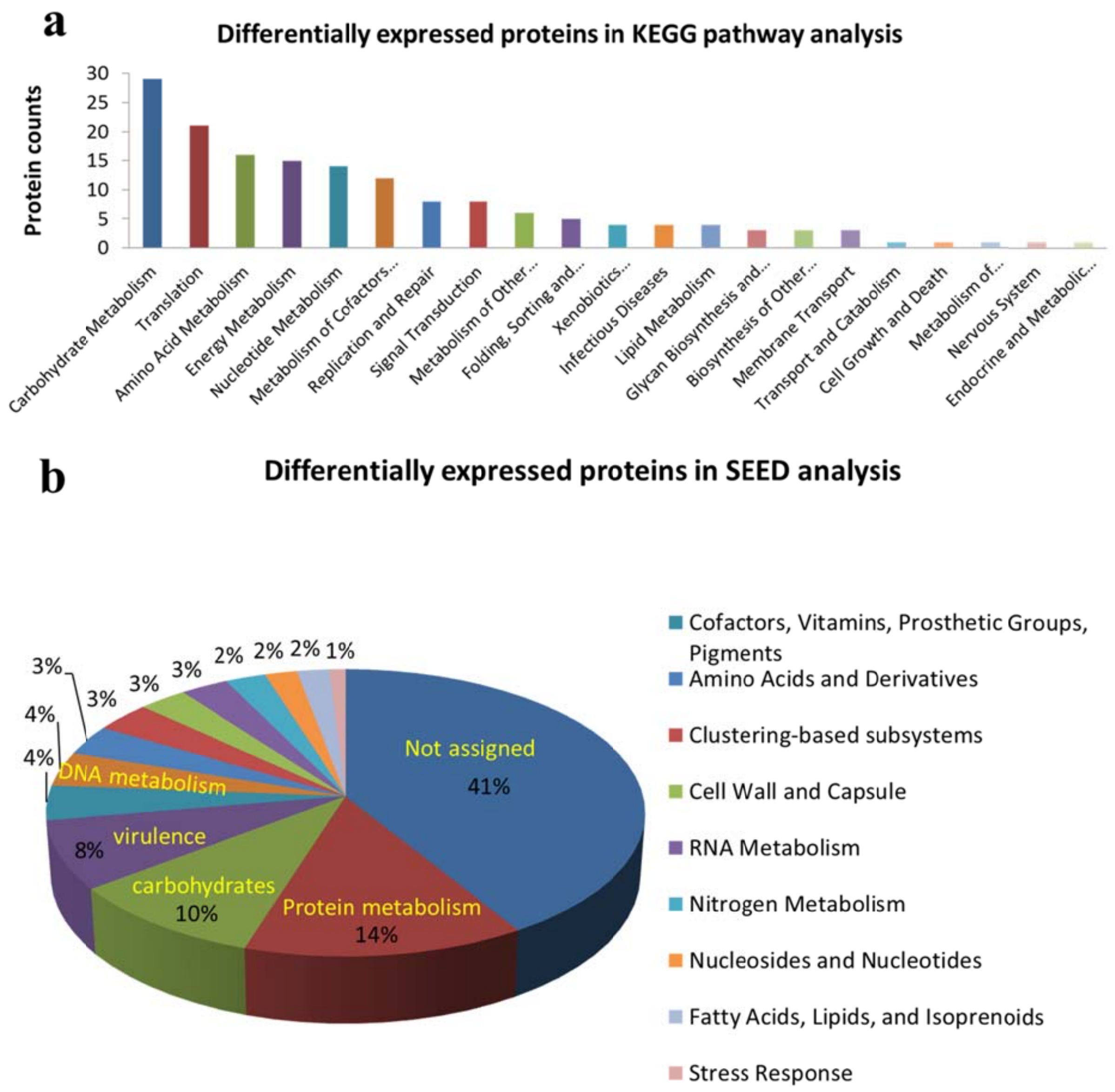
a
Differentially expressed proteins in KEGG pathway analysis
Protein counts
30
25
20
15
10
5
0
Carbohydrate Metabolism
Translation
Amino Acid Metabolism
Energy Metabolism
Nucleotide Metabolism
Metabolism of Cofactors...
Replication and Repair
Signal Transduction
Metabolism of Other...
Folding, Sorting and...
Xenobiotics...
Infectious Diseases
Lipid Metabolism
Glycan Biosynthesis and...
Biosynthesis of Other...
Membrane Transport
Transport and Catabolism
Cell Growth and Death
Metabolism of...
Nervous System
Endocrine and Metabolic...
b
Differentially expressed proteins in SEED analysis
Not assigned
41%
Protein metabolism
14%
carbohydrates
10%
virulence
8%
DNA metabolism
4%
4%
3%
3%
3%
3%
2%
2%
2%
1%
Cofactors, Vitamins, Prosthetic Groups, Pigments
Amino Acids and Derivatives
Clustering-based subsystems
Cell Wall and Capsule
RNA Metabolism
Nitrogen Metabolism
Nucleosides and Nucleotides
Fatty Acids, Lipids, and Isoprenoids
Stress Response

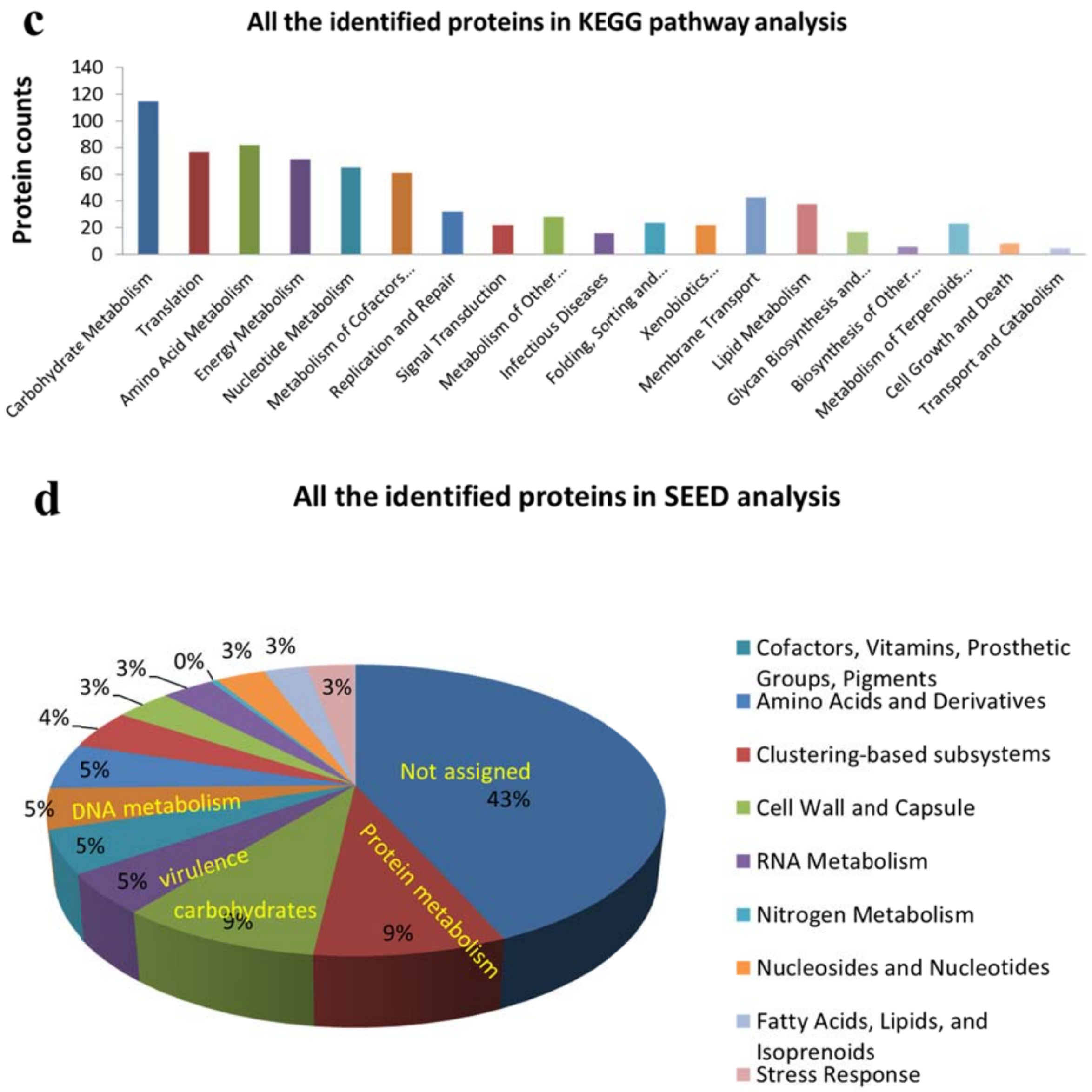

Figure S2: KEGG pathway (a) and SEED analysis (b) for the differentially expressed proteins in SIPI-8294/Oxa treatment group; KEGG pathway (c) and SEED analysis (d) for all the identified proteins in SIPI-8294/Oxa treatment group

a

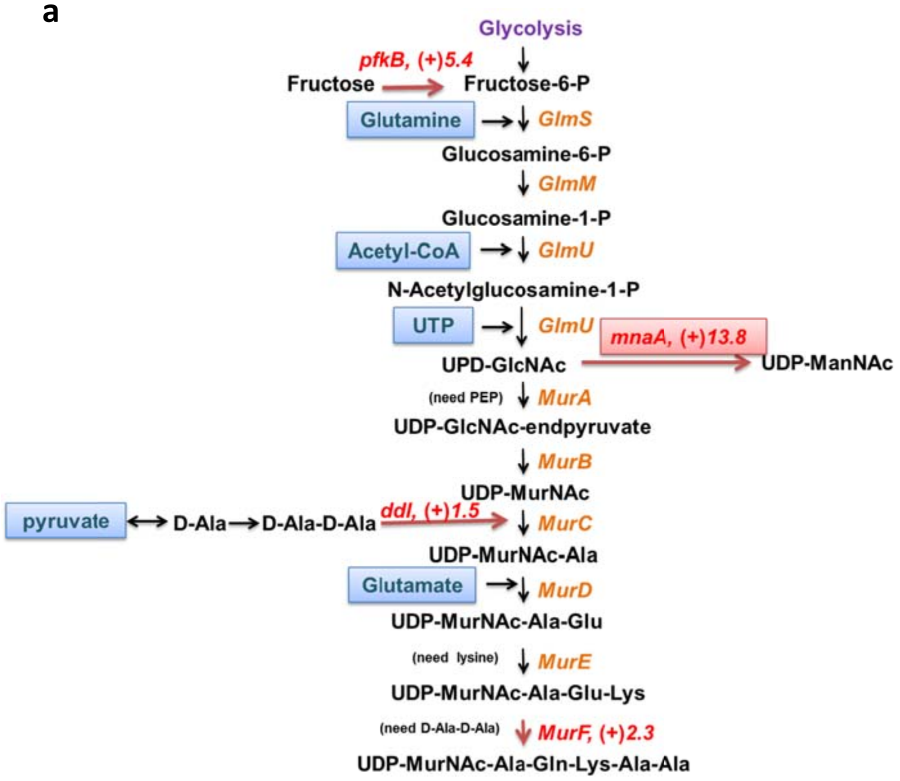
Glycolysis
pfkB, (+)5.4
Fructose
Fructose-6-P
Glutamine
GlmS
Glucosamine-6-P
GlmM
Glucosamine-1-P
Acetyl-CoA
GlmU
N-Acetylglucosamine-1-P
UTP
GlmU
mnaA, (+)13.8
UPD-GlcNAc
UDP-ManNAc
(need PEP)
MurA
UDP-GlcNAc-endpyruvate
MurB
UDP-MurNAc
pyruvate
D-Ala
D-Ala-D-Ala
ddl, (+)1.5
MurC
UDP-MurNAc-Ala
Glutamate
MurD
UDP-MurNAc-Ala-Glu
(need lysine)
MurE
UDP-MurNAc-Ala-Glu-Lys
(need D-Ala-D-Ala)
MurF, (+)2.3
UDP-MurNAc-Ala-Gln-Lys-Ala-Ala

b

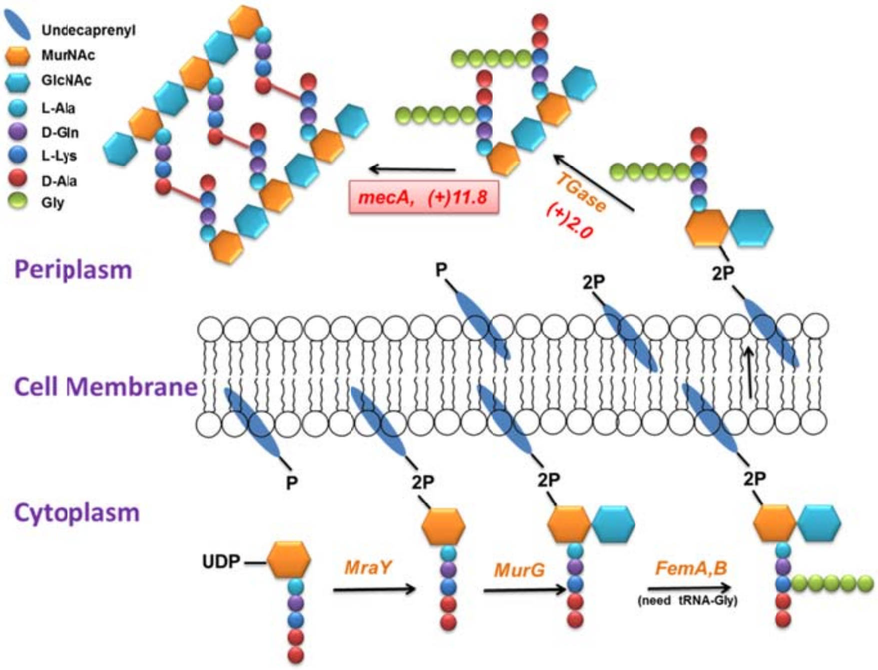
Undecaprenyl
MurNAc
GlcNAc
L-Ala
D-Gln
L-Lys
D-Ala
Gly
mecA, (+)11.8
TGase
(+)2.0
Periplasm
P
2P
2P
Cell Membrane
Cytoplasm
P
2P
2P
2P
UDP
MraY
MurG
FemA,B
(need tRNA-Gly)

Figure S3: The enzymes involved in cell wall biosynthesis: (a) The synthesis of UDP-MurNAc-Ala-Gln-Lys-Ala-Ala which is the blocks for building cell wall. (b) The process of transfering the blocks from cytoplasm to periplasm, and the following transpeptidation steps for the cell wall synthesis. The metabolites in blue boxes are the chemical components needed for the reaction step. The enzymes are labeled by their corresponding gene names. The symbol of (+) stands for up-regulated; number behind the gene name is the fold change. The enzymes with up-regulated fold changes more than 10 are highlighted in red boxes.

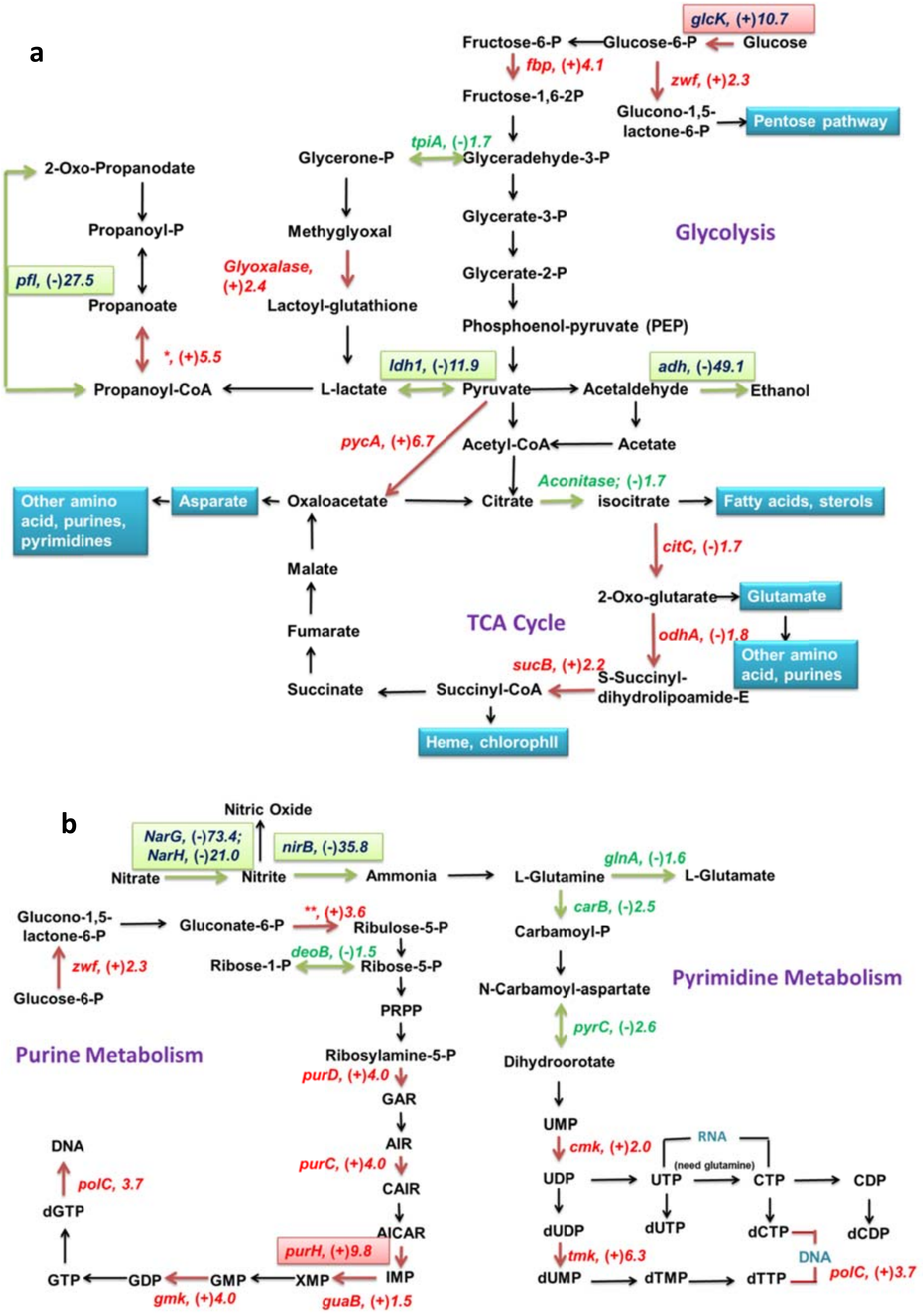
a
glcK, (+)10.7
Fructose-6-P
Glucose-6-P
Glucose
fbp, (+)4.1
zwf, (+)2.3
Fructose-1,6-2P
Glucono-1,5-lactone-6-P
Pentose pathway
tpiA, (-)1.7
Glycerone-P
Glyceradehyde-3-P
2-Oxo-Propanodate
Propanoyl-P
Methylglyoxal
Glycerate-3-P
Glycolysis
pfl, (-)27.5
Glyoxalase, (+)2.4
Glycerate-2-P
Propanoate
Lactoyl-glutathione
Phosphoenol-pyruvate (PEP)
*, (+)5.5
ldh1, (-)11.9
adh, (-)49.1
Propanoyl-CoA
L-lactate
Pyruvate
Acetaldehyde
Ethanol
pycA, (+)6.7
Acetyl-CoA
Acetate
Aconitase; (-)1.7
Other amino acid, purines, pyrimidines
Asparate
Oxaloacetate
Citrate
isocitrate
Fatty acids, sterols
citC, (-)1.7
Malate
2-Oxo-glutarate
Glutamate
TCA Cycle
Fumarate
odhA, (-)1.8
Other amino acid, purines
sucB, (+)2.2
S-Succinyl-dihydrolipoamide-E
Succinate
Succinyl-CoA
Heme, chlorophll
b
Nitric Oxide
NarG, (-)73.4; NarH, (-)21.0
nirB, (-)35.8
Nitrate
Nitrite
Ammonia
L-Glutamine
glnA, (-)1.6
L-Glutamate
Glucono-1,5-lactone-6-P
Gluconate-6-P
**, (+)3.6
Ribulose-5-P
carB, (-)2.5
Carbamoyl-P
zwf, (+)2.3
deoB, (-)1.5
Ribose-1-P
Ribose-5-P
Glucose-6-P
N-Carbamoyl-aspartate
Pyrimidine Metabolism
PRPP
pyrC, (-)2.6
Purine Metabolism
Ribosylamine-5-P
Dihydroorotate
purD, (+)4.0
GAR
UMP
DNA
AIR
cmk, (+)2.0
RNA
(need glutamine)
polC, 3.7
purC, (+)4.0
UDP
UTP
CTP
CDP
CAIR
dGTP
dUTP
dUDP
dCTP
dCDP
AICAR
purH, (+)9.8
DNA
tmk, (+)6.3
GTP
GDP
GMP
XMP
IMP
dUMP
dTMP
dTTP
polC, (+)3.7
gmk, (+)4.0
guaB, (+)1.5

Figure S4: (a) The enzymes differentially expressed in carbohydrate metabolism pathways; * stands for AMP-dependent synthetase and ligase; (b) the enzymes differentially expressed in nitrogen and purine/pyrimidine metabolism pathways; ** stands for 6-phosphogluconate dehydrogenase, decarboxylating (EC 1.1.1.44). The enzymes are labeled by their corresponding gene names. The symbol of (+) stands for up-regulated and the symbol of (-) stands for down-regulated; number behind the gene name is the fold change. The enzymes with up-regulated and down-regulated fold changes more than 10 are highlighted in red and green boxes, respectively.

Table S1: The differentially expressed proteins in different treatment groups

Uniprot_AC	gene	protein	fold change					pathway
			8294+Oxa	Ery+Oxa	SIPI-8294	Oxa	Ery	
Carbohydrates								
A5IQX6	SaurJH9_0797	Glyceraldehyde-3-phosphate dehydrogenase (EC 1.2.1.12)	-	-	-	(+)1.5		
A5IT75	SaurJH9_1605	Glucokinase (EC 2.7.1.2)	(+)10.7 (Induction)	(+)11.8 (Induction)	-	-	(+)7.3 (Induction)	Amino sugar and nucleotide sugar metabolism; Starch and sucrose metabolism; Glycolysis / Gluconeogenesis; Galactose metabolism
A5ITV7	pckA	Phosphoenolpyruvate carboxykinase [ATP] (EC 4.1.1.49)	(+)4.0	(+)3.3	-	-	-	Citrate cycle (TCA cycle); Carbon fixation in photosynthetic organisms; Glycolysis / Gluconeogenesis; Pyruvate metabolism
A5ITM5	SaurJH9_1758	Malate dehydrogenase (Oxaloacetate-decarboxylating) (EC 1.1.1.38)	(-)1.7	-	-	-	-	Two-component system; Pyruvate metabolism
A6QFW9	pycA	Pyruvate carboxylase (EC 6.4.1.1)	(+)6.7	ND	(+)12.4	-	-	Citrate cycle (TCA cycle); Carbon fixation pathways in prokaryotes; Pyruvate metabolism
A5IT65	gcvT	Aminomethyltransferase (EC 2.1.2.10)	-	-	(+)1.5	-	-	Glycine, serine and threonine metabolism; Nitrogen metabolism; One carbon pool by folate
A5IQE2	SaurJH9_0611	Phosphotransacetylase (EC 2.3.1.8)	(-)1.6	-	-	-	-	Carbon fixation pathways in prokaryotes; Taurine and hypotaurine metabolism; Methane metabolism; Propanoate metabolism; Pyruvate metabolism
A5IP09	deoB	Phosphopentomutase (EC 5.4.2.7)	(-)1.5	-	-	-	-	Pentose phosphate pathway; Purine metabolism
A5IUZ5	SaurJH9_2237	Acetolactate synthase, large subunit (EC 2.2.1.6)	ND	ND	ND	(+)26.1	-	Butanoate metabolism; Valine, leucine and isoleucine biosynthesis; Pantothenate and CoA biosynthesis
A5IQC5	SaurJH9_0594	Hexulose-6-phosphate isomerase (EC 5.-.-.-)	-	(+)3.2	-	-	(+)3.8	Methane metabolism; Pentose and glucuronate interconversions; Pentose phosphate pathway
A5IPA9	ldh1	L-lactate dehydrogenase 1 (L-LDH 1) (EC 1.1.1.27)	(-)11.9	(-)3.5	-	(+)1.6	(-)3.5	Cysteine and methionine metabolism; Glycolysis / Gluconeogenesis; Propanoate metabolism; Pyruvate metabolism
A5IW27	ldh2	L-lactate dehydrogenase 2 (L-LDH 2) (EC 1.1.1.27)	(-)1.9	(-)2.2	-	-	(-)2.0	Cysteine and methionine metabolism; Glycolysis / Gluconeogenesis; Propanoate metabolism; Pyruvate metabolism
A5ISU5	odhA	2-oxoglutarate dehydrogenase E1 component (EC 1.2.4.2)	(+)1.8	-	-	-	-	Tryptophan metabolism; Citrate cycle (TCA cycle); Lysine degradation
A5IWG6	SaurJH9_2765	6-phosphogluconate dehydrogenase, decarboxylating (EC 1.1.1.44)	(+)3.6	(+)3.0	-	-	-	Glutathione metabolism; Pentose phosphate pathway
A5IT39	gnd	6-phosphogluconate dehydrogenase, decarboxylating (EC 1.1.1.44)	-	(+)1.8	-	-	-	Glutathione metabolism; Pentose phosphate pathway
A5IT33	SaurJH9_1562	Glucose-6-phosphate 1-dehydrogenase (EC 1.1.1.49)	(+)2.3	-	-	-	(+)1.9	Glutathione metabolism; Pentose phosphate pathway
A5IQ53	prs	Ribose-phosphate pyrophosphokinase (RPPK) (EC 2.7.6.1)	-	(-)1.7	-	-	-	Pentose phosphate pathway; Purine metabolism
A5IU17	fumC	Fumarate hydratase class II (Fumarase C) (EC 4.2.1.2)	-	-	(+)1.7	-	-	Citrate cycle (TCA cycle); Carbon fixation pathways in prokaryotes; Pathways in cancer; Renal cell carcinoma
D2N740	sucB	Dihydrolipoyllysine-residue succinyltransferase (EC 2.3.1.61)	(+)2.2	-	-	-	-	Citrate cycle (TCA cycle); Lysine degradation
D9RHY0	citC	Isocitrate dehydrogenase [NADP] (EC 1.1.1.42)	(+)1.7	-	-	-	-	Citrate cycle (TCA cycle); Carbon fixation pathways in prokaryotes; Glutathione metabolism; Peroxisome
A5IVU3	fbp	Fructose-1,6-bisphosphatase class 3 (FBPase class 3) (EC 3.1.3.11)	(+)4.1	-	-	-	(+)2.1	Fructose and mannose metabolism; Carbon fixation in photosynthetic organisms; Glycolysis / Gluconeogenesis; Pentose phosphate pathway
A5IUS0	SaurJH9_2161	Fructose-bisphosphate aldolase (EC 4.1.2.13)	-	(+)1.6	-	-	-	Fructose and mannose metabolism; Carbon fixation in photosynthetic organisms; Glycolysis / Gluconeogenesis; Pentose phosphate pathway
A5IRA6	SaurJH9_0928	HAD-superfamily subfamily IIA hydrolase like protein	-	(+)2.9	-	-	-	
A5IW34	SaurJH9_2631	AMP-dependent synthetase and ligase	(+)5.5	-	-	-	-	Carbon fixation pathways in prokaryotes; Glycolysis / Gluconeogenesis; Propanoate metabolism; Pyruvate metabolism
A8Z0I5	pfl	Formate C-acetyltransferase (EC 2.3.1.54)	(-)27.5	(-)5.3	-	-	(-)7.2	Butanoate metabolism; Propanoate metabolism; Pyruvate metabolism
A5IUU9	mtlD	Mannitol-1-phosphate 5-dehydrogenase (EC 1.1.1.17)	(-)1.8	-	-	-	-	Fructose and mannose metabolism
A5ISN3	SaurJH9_1412	Aconitase (EC 4.2.1.3)	(-)1.7	-	-	-	-	Citrate cycle (TCA cycle); Carbon fixation pathways in prokaryotes
E5QRL3	HMPREF0772_11158	Sucrose-6-phosphate hydrolase (EC 3.2.1.26)	(+)2.9	(+)2.2	-	-	-	Starch and sucrose metabolism; Galactose metabolism
A5IUI6	SaurJH9_2077	PfkB domain protein	(+)5.4	ND	ND	ND	-	Amino sugar and nucleotide sugar metabolism; Fructose and mannose metabolism; Starch and sucrose metabolism
Cofactors, Vitamins, Prosthetic Groups, Pigments;								
A5IQD4	SaurJH9_0603	Phosphomethylpyrimidine kinase	(+)2.7	-	-	-	-	Vitamin B6 metabolism
A6QF28	NWMN_0688	ABC transporter, ATP-binding protein	-	-	-	(+)3.5	-	
E5QRF7	lipA	Lipoyl synthase (EC 2.8.1.8)	(+)6.3	(+)6.9	-	-	(+)4.1	Lipoic acid metabolism

E5QS29	nadE	NH(3)-dependent NAD(+) synthetase (EC 6.3.1.5)	(+)8.4 (Induction)	(+)5.5 (Induction)	-	-	-	Nicotinate and nicotinamide metabolism
A5IQ72	pdxS	Pyridoxal biosynthesis lyase PdxS (EC 4.-.-.-)	(-)2.0	-	-	(-)1.5	-	Vitamin B6 metabolism
E5QW19	panC	Pantothenate synthetase (PS) (EC 6.3.2.1)	(+)3.6	(+)2.8	-	(+)1.9	-	beta-Alanine metabolism; Pantothenate and CoA biosynthesis
A5ITZ8	hemH	Ferrochelatase (EC 4.99.1.1)	ND	ND	-	-	(+)2.0	Porphyrin and chlorophyll metabolism
A5ITZ9	hemE	Uroporphyrinogen decarboxylase (UPD) (URO-D) (EC 4.1.1.37)	(+)1.9	-	-	-	-	Porphyrin and chlorophyll metabolism
A5ITJ3	SaurJH9_1726	Delta-aminolevulinic acid dehydratase (EC 4.2.1.24)	(+)1.7	-	-	-	-	Porphyrin and chlorophyll metabolism
A5ITL2	coaE	Dephospho-CoA kinase (EC 2.7.1.24)	(+)2.7	-	-	(+)1.8	-	Pantothenate and CoA biosynthesis
Virulence									
A5IUH6	groL	60 kDa chaperonin (GroEL protein)	(+)2.0	(+)1.6	-	-	-	RNA degradation;
A8Z071	spsB	Sec family Type I general secretory pathway S26 family signal peptidase I (EC 3.4.21.89)	(+)1.9	-	-	-	-	Protein export
A5IPF1	SaurJH9_0267	Membrane protein-like protein	ND	ND	(-)2.6 (Repression)	(-)22.3 (Repression)	ND	
A5IRU3	fmtA	protein FmtA	(+)4.5 (Induction)	ND	-	-	-	
A5IU13	SaurJH9_1901	Transcriptional regulator, XRE family	(-)2.5	(-)1.5	-	(-)1.8	-	
D1GTW3	clfB	Fibrinogen and keratin-10 binding surfaceanchored protein	(-)3.6	(-)2.5	(-)2.7	-	-	
A7WX60	spa	Immunoglobulin G binding protein A	ND	ND	-	(-)4.2	-	
A5IV44	SaurJH9_2287	Acriflavin resistance protein	(+)2.2	ND	-	-	-	
Q6GDU5	fnbA	Fibronectin-binding protein A	(-)4.0	(-)2.2	-	-	-	Bacterial invasion of epithelial cells
A6U5C6	blaZ	β-lactamase (EC 3.5.2.6)	ND	ND	ND	(+)12.5 (Induction)	ND	β-Lactam resistance
D8HDS7	SAOV_2224c	Ferrichrome ABC transporter lipoprotein	-	(-)2.1	-	-	-	ABC transporters
A5IS94	gmk	Guanylate kinase (EC 2.7.4.8)	(+)4.0 (Induction)	(+)4.3 (Induction)	-	-	-	Purine metabolism
A5IQX8	tpiA	Triosephosphate isomerase (TIM) (EC 5.3.1.1)	(-)1.7	-	-	-	-	Fructose and mannose metabolism; Carbon fixation in photosynthetic organisms; Glycolysis / Gluconeogenesis
A5IQU0	SaurJH9_0760	Periplasmic binding protein	(-)4.7	(-)4.4	-	-	-	ABC transporters
D8HHN1	SAOV_0999c	Autolysin	(-)2.3	(-)4.2	-	(-)2.5	-	
A5INS0	mecA	Penicillin binding protein 2a (EC 2.4.1.129)	(+)11.8	(+)42.0	ND	(+)145.1	ND	β-Lactam resistance
A5IVD4	SaurJH9_2377	Multidrug resistance efflux pump-like protein	-	-	-	(+)1.6	-	
A5IRB3	SaurJH9_0935	DltD, central domain protein	(+)4.6	-	-	-	-	Staphylococcus aureus infection
A5INS9	SaurJH9_0040 SaurJH9_1714	Streptomycin 3''-adenylyltransferase (EC 2.7.7.47)	(+)2.9	-	-	-	-	
A5IPU8	rpsR	30S ribosomal protein S18	(-)2.0	(-)1.7	-	-	-	Ribosome
Q6GIX3	mco	Multicopper oxidase mco (EC 1.-.-.-)	(+)4.0 (Induction)	ND	ND	ND	ND	
Protein Metabolism									
A5ISH6	miaB	(Dimethylallyl)adenosine tRNA methylthiotransferase MiaB (EC 2.-.-.-)	(+)12.8	ND	-	-	-	
A5ITA4	SaurJH9_1634	RNA modification enzyme, MiaB family (EC 1.3.1.74)	(+)2.4	(+)2.0	-	-	-	
A5IT57	efp	Elongation factor P (EF-P)	-	-	(-)2.3	-	-	
E5QRA6	clpB	ATP-dependent chaperone protein ClpB	(+)4.3	(+)3.0	-	(+)2.8	-	
A5IUH6	groL	60 kDa chaperonin (GroEL protein)	(+)2.0	(+)1.6	-	-	-	RNA degradation
A6QI23	prsA	Foldase protein PrsA (EC 5.2.1.8)	(+)9.6	(+)4.3	(+)1.7	-	-	
E5QVW1	asnS	AsparagineNDtRNA ligase (EC 6.1.1.22)	(+)1.7	-	-	-	-	Aminoacyl-tRNA biosynthesis
A5IT95	era	GTPase Era	(+)2.6	-	(-)1.9	-	-	
A5ITG8	obg	GTPase obg (GTP-binding protein obg)	(+)1.7	(+)1.6	-	-	-	
A5IT03	der	GTPase Der (GTP-binding protein EngA)	-	-	-	(+)1.7	-	
A5ITC4	SaurJH9_1654	Nitric-oxide synthase (EC 1.14.13.39)	(+)2.5	-	-	-	-	
A5IPU4	SaurJH9_0411	GTP-binding protein YchF	(-)1.5	-	-	-	-	
A5IV31	rplB	50S ribosomal protein L2	(-)1.9	-	(-)1.8	-	-	Ribosome
A5IV33	rplD	50S ribosomal protein L4	-	(+)2.1	-	-	-	Ribosome
A5IV22	rplE	50S ribosomal protein L5	-	(+)1.8	-	-	(+)1.7	Ribosome
A5IV02	rplM	50S ribosomal protein L13	(-)1.8	-	-	-	-	Ribosome
A6QEI9	rplK	50S ribosomal protein L11	-	(+)1.8	-	-	(+)2.0	Ribosome
A5IV15	rplO	50S ribosomal protein L15	-	(+)1.7	-	-	-	Ribosome
A5IV24	rplN	50S ribosomal protein L14	(-)2.1	-	-	-	-	Ribosome
A5IV07	rplQ	50S ribosomal protein L17	-	(+)2.2	-	-	(+)2.1	Ribosome
A5IV27	rplP	50S ribosomal protein L16	(-)1.8	-	-	-	-	Ribosome
A5ITH1	rplU	50S ribosomal protein L21	-	-	(-)2.0	-	-	Ribosome
A5ITK3	rplT	50S ribosomal protein L20	-	-	(-)1.6	-	(-)1.6	Ribosome

A5IV32	rplW	50S ribosomal protein L23	(-)3.3	-	-	-	-	Ribosome
A5IV29	rplV	50S ribosomal protein L22	(-)1.7	-	-	-	-	Ribosome
A5ITG9	rpmA	50S ribosomal protein L27	(-)2.0	-	-	(-)2.0	-	Ribosome
A5IV23	rplX	50S ribosomal protein L24	(-)2.8	-	-	-	(+)2.0	Ribosome
A5IV11	rpmJ	50S ribosomal protein L36		(+)3.6	-	(+)3.8	-	Ribosome
A8Z216	rpmG2	50S ribosomal protein L33 2	(+)5.4	(+)6.1	-	-	-	Ribosome
A5IR09	SaurJH9_0830	Thioredoxin domain	(-)1.7	-	-	-	-	
E5QS12	HMPREF0772_11212	Uncharacterized protein	-	-	(+)1.6	-	-	
A5IQA0	rpsG	30S ribosomal protein S7	-	(+)1.7	-	-	-	Ribosome
A5IPU6	rpsF	30S ribosomal protein S6	(-)3.1	(-)1.8	-	-	-	Ribosome
A5ITP2	rpsD	30S ribosomal protein S4	-	(+)1.8	-	-	-	Ribosome
A5IV09	rpsK	30S ribosomal protein S11	-	(+)2.0	-	-	-	Ribosome
A5IV35	rpsJ	30S ribosomal protein S10	(+)2.6	(+)3.6	-	-	-	Ribosome
A8Z324	rpsI	30S ribosomal protein S9	(+)2.7	(+)3.4	-	-	(+)2.7	Ribosome
A5IV20	rpsH	30S ribosomal protein S8	-1.6	-	-	-	-	Ribosome
A5IV10	rpsM	30S ribosomal protein S13	(+)2.4	(+)3.6	-	-	-	Ribosome
A5IQ99	rpsL	30S ribosomal protein S12	(+)5.8	(+)5.9	-	-	-	Ribosome
A5IV30	rpsS	30S ribosomal protein S19	-	-	-	-	(-)2.1	Ribosome
A5IPU8	rpsR	30S ribosomal protein S18	(-)2.0	(-)1.7	-	-	-	Ribosome
A5IT04	SaurJH9_1533	SSU ribosomal protein S1P	(+)1.7	-	-	-	-	Ribosome
A5ISP8	SaurJH9_1427	Peptidase M42 family protein	-	-	-	-	(+)1.6	
RNA Metabolism								
A5IQ56	SaurJH9_0525	Transcription-repair coupling factor	(+)7.2 (Induction)	(+)5.8 (Induction)	-	(+)4.8	-	Nucleotide excision repair
A5IQY4	rnr	Ribonuclease R (EC 3.1.13.1)	(+)1.7	-	-	-	-	RNA degradation
A5ISH6	miaB	(Dimethylallyl)adenosine tRNA methylthiotransferase MiaB (EC 2.-.-.-)	(+)12.8	ND	-	-	-	
A5IR24	SaurJH9_0846	SUF system FeS assembly protein, NifU family	(+)4.5	(+)3.3	-	-	-	
E5QVT7	ypdA	Bacillithiol system thiol disulfide oxidoreductase, YpdA family (EC 1.8.-.-)	-	(+)4.8	-	(+)1.9	-	Pyrimidine metabolism
E5QR89	HMPREF0772_11114	DEAD/DEAH box helicase	(-)1.5	-	-	-	-	RNA degradation
A5ITF1	SaurJH9_1684	Transcriptional regulator, BadM/Rrf2 family	-	-	(+)1.7	-	-	
D8HBV7	queA	S-adenosylmethionine:tRNA ribosyltransferase-isomerase (EC 2.4.99.17)	-	(+)5.1	(-)1.7	(+)1.5	-	
A5IRD2	SaurJH9_0954	Peptidylprolyl isomerase (EC 5.2.1.8)	(-)3.5	(-)1.6	-	-	-	
A5IS95	rpoZ	DNA-directed RNA polymerase subunit omega (EC 2.7.7.6)	-	(-)2.2	-	-	-	RNA polymerase; Purine metabolism; Pyrimidine metabolism
A5ITD5	greA	Transcription elongation factor GreA	-	-	-	-	(+)1.8	
Amino Acids and Derivatives								
A5IT44	SaurJH9_1574	Branched-chain alpha-keto acid dehydrogenase E2 component (EC 2.3.1.168)	(+)2.9	-	-	-	-	Valine, leucine and isoleucine degradation
A5IVV8	SaurJH9_2554	2-hydroxymethylglutarate dehydratase (EC 4.2.1.-)	ND	ND	-	(-)4.5	-	Cysteine and methionine metabolism; Glycine, serine and threonine metabolism
A6QFC7	sufS	Cysteine desulfurase SufS subfamily protein	-	-	-	(+)1.9	-	Thiamine metabolism
A5IST2	lysA	Diaminopimelate decarboxylase (DAP decarboxylase) (DAPDC) (EC 4.1.1.20)	(+)5.5 (Induction)	-	-	-	-	Lysine biosynthesis
A5IVB3	hutU	Urocanate hydratase (Urocanase) (EC 4.2.1.49)	ND	ND	(+)8.1 (Induction)	ND	ND	Histidine metabolism
D9RNE5	rocD	Ornithine aminotransferase (OAT) (EC 2.6.1.13)	-	(-)2.2	-	-	-	Arginine and proline metabolism
A5IVY0	rocA	1-pyrroline-5-carboxylate dehydrogenase (P5C dehydrogenase) (EC 1.2.1.88)	(+)1.6	-	-	-	-	Alanine, aspartate and glutamate metabolism; Arginine and proline metabolism
A5IQA4	SaurJH9_0573	2-amino-3-ketobutyrate coenzyme A ligase (EC 2.3.1.29)	(+)2.7	(+)2.9	-	-	-	Glycine, serine and threonine metabolism
A5IV71	ureC	Urease subunit alpha (EC 3.5.1.5)	(+)3.0	(+)1.5	(+)3.4	(+)2.1	-	Arginine and proline metabolism; Purine metabolism
A5IV72	ureE	Urease accessory protein UreE	-	(+)4.0 (Induction)	(+)4.2	-	-	
A5IV74	ureG	Urease accessory protein UreG	(+)13.9 (Induction)	ND	(+)2.5	(+)2.1	ND	
A5IUA5	SaurJH9_1994	Aminotransferase (EC 2.6.1.-)	(+)2.3	-	-	-	-	
Clustering-based subsystems;								
A5IRX7	SaurJH9_1150	UPF0356 protein SaurJH9_1150	(-)3.1	(-)2.9	-	(-)1.7	(-)3.5	
A5INQ8	purA	Adenylosuccinate synthetase (AMPSase) (AdSS) (EC 6.3.4.4)	(-)1.9	-	-	-	-	
A5INQ7	SaurJH9_0016	Primary replicative DNA helicase (EC 3.6.1.-)	(+)14.7	ND	ND	-	-	DNA replication; Alanine, aspartate and glutamate metabolism; Purine metabolism
A5IRX8	def	Peptide deformylase (PDF) (EC 3.5.1.88)	(-)1.6	-	-	-	-	
A5IS65	rsmH	Ribosomal RNA small subunit methyltransferase H (EC 2.1.1.199)	(+)3.1	(+)2.9	-	-	-	
D9RL57	tig	Trigger factor (TF) (EC 5.2.1.8) (PPIase)	(-)1.9	-	-	-	-	
A5IT95	era	GTPase Era	(+)2.6	-	(-)1.9	-	-	

A5IQ64	ftsH	ATP-dependent zinc metalloprotease FtsH (EC 3.4.24.-)	-	(-)2.2	-	-	(-)1.6	
Fatty Acids, Lipids, and Isoprenoids;								
A5ITM4	accD	Acetyl-coenzyme A carboxylase carboxyl transferase subunit beta (EC 6.4.1.2)	-	-	-	-	(+)1.8	Fatty acid biosynthesis; Carbon fixation pathways in prokaryotes; Propanoate metabolism; Pyruvate metabolism
A5ISB7	acpP	Acyl carrier protein (ACP)	(-)2.9	(-)2.1	-	-	-	
A5IQF9	adh	Alcohol dehydrogenase	(-)49.1 (Repression)	(-)13.1	-	(+)3.2	(-)4.0	Tyrosine metabolism; Fatty acid metabolism; Glycolysis / Gluconeogenesis;
A5IT02	gpsA	Glycerol-3-phosphate dehydrogenase [NAD(P)+] (EC 1.1.1.94)	(+)6.6	(+)5.0	-	-	-	Glycerophospholipid metabolism
A5ISB1	SaurJH9_1285	Dak phosphatase	(+)1.6	-	-	-	-	
Nucleosides and Nucleotides								
A5IS85	pyrB	Aspartate carbamoyltransferase (EC 2.1.3.2)	ND	ND	(-)2.3	(-)2.4	-	Alanine, aspartate and glutamate metabolism; Pyrimidine metabolism
A5IS86	pyrC	Dihydroorotase (DHOase) (EC 3.5.2.3)	(-)2.6	(-)1.7	-	-	(-)1.9	Pyrimidine metabolism
A5IRW1	purD	PhosphoribosylamineNDglycine ligase (EC 6.3.4.13)	(+)4.0 (Induction)	ND	ND	ND	ND	Purine metabolism
D8HHP3	SAOV_1011	Phosphoribosylaminoimidazole-succinocarboxamide synthase	(+)4.0 (Induction)	ND	ND	ND	ND	Purine metabolism
A5ITF9	apt	Adenine phosphoribosyltransferase (APRT) (EC 2.4.2.7)	-	(+)2.4	-	-	(+)2.1	Purine metabolism
A5IPW9	guaB	Inosine-5'-monophosphate dehydrogenase (EC 1.1.1.205)	(+)1.5	-	-	-	-	Purine metabolism
Cell Wall and Capsule;								
Q6GEW4	mnaA	UDP-N-acetylglucosamine 2-epimerase (EC 5.1.3.14)	(+)13.8	ND	-	-	ND	Amino sugar and nucleotide sugar metabolism
A5IPC6	SaurJH9_0242	CDP-glycerol:poly(Glycerophosphate) glycerophosphotransferase	(-)2.9	(-)2.8	-	-	(-)2.4	
A5IU40	mgt	Monofunctional glycosyltransferase (MGT) (EC 2.4.-.-)	ND	ND	(+)7.5 (Induction)	ND	ND	Peptidoglycan biosynthesis
A5INS0	mecA	Peptidoglycan glycosyltransferase (EC 2.4.1.129)	(+)11.8	(+)42.0	ND	(+)145.1	ND	beta-Lactam resistance
A5IUW0	SaurJH9_2202	UTPNDglucose-1-phosphate uridylyltransferase	-	(+)2.4	-	-	-	
A5IQU2	murB	UDP-N-acetylenolpyruvoylglucosamine reductase (EC 1.3.1.98)	ND	ND	(+)3.1	ND	ND	Amino sugar and nucleotide sugar metabolism; Peptidoglycan biosynthesis
E5QR87	ddl	D-alanineNDD-alanine ligase (EC 6.3.2.4)	(+)1.5	-	-	(+)1.6	-	D-Alanine metabolism; Peptidoglycan biosynthesis
A5IS69	murD	UDP-N-acetylmuramoylalanineNDD-glutamate ligase (EC 6.3.2.9)	-	-	-	(+)1.6	-	D-Glutamine and D-glutamate metabolism; Peptidoglycan biosynthesis
A5ISB7	acpP	Acyl carrier protein (ACP)	(-)2.9	(-)2.1	-	-	-	
A5IRB0	dltA	D-alanineNDpoly(phosphoribitol) ligase subunit 1 (EC 6.1.1.13)	-	-	-	-	(+)1.5	D-Alanine metabolism; Staphylococcus aureus infection
A5IRB3	SaurJH9_0935	DltD, central domain protein	(+)4.6	-	-	-	-	Staphylococcus aureus infection
DNA Metabolism;								
A5IQ56	SaurJH9_0525	Transcription-repair coupling factor	(+)7.2 (Induction)	(+)5.8 (Induction)	-	(+)4.8	-	Nucleotide excision repair
A6QGJ5	mutL	DNA mismatch repair protein MutL	(+)4.9	ND	ND	-	ND	Mismatch repair
A5ISH9	mutS	DNA mismatch repair protein MutS	-	-	-	(+)1.6	-	Mismatch repair
A5INQ7	SaurJH9_0016	Primary replicative DNA helicase (EC 3.6.1.-)	(+)14.7	ND	ND	-	-	DNA replication; Alanine, aspartate and glutamate metabolism; Purine metabolism
A5IQW3	uvrA	UvrABC system protein A (UvrA protein) (Excinuclease ABC subunit A)	-	(-)1.6	-	-	(-)2.9	Nucleotide excision repair
E5QS79	uvrB	UvrABC system protein B (Protein UvrB) (Excinuclease ABC subunit B)	(+)5.3 (Induction)	ND	(+)3.1	-	-	Nucleotide excision repair
A5ISX1	SaurJH9_1500	5'-3' exonuclease	-	-	-	(+)1.8	-	
A5INP3	SaurJH9_0002	DNA polymerase III subunit beta (EC 2.7.7.7)	(+)1.5	-	-	-	-	DNA replication; Mismatch repair; Purine metabolism; Pyrimidine metabolism
A5ISE8	polC	DNA polymerase III polC-type (PolIII) (EC 2.7.7.7)	(+)3.7	-	-	-	-	DNA replication; Mismatch repair; Purine metabolism; Pyrimidine metabolism
A5INP2	dnaA	Chromosomal replication initiator protein DnaA	-	-	(-)2.1	-	-	Two-component system
A5IU72	SaurJH9_1960	ATP-dependent DNA helicase PcrA (EC 3.6.1.-)	(+)1.6	-	-	-	-	Nucleotide excision repair; Mismatch repair
A5ISY5	SaurJH9_1514	DnaQ family exonuclease/DinG family helicase, putative	-	-	-	(+)3.0	-	
A5IQ79	SaurJH9_0548	DNA repair protein radA	-	-	(+)1.9	(+)2.3	-	
A5IT48	SaurJH9_1578	DNA repair protein RecN (Recombination protein N)	-	-	(+)3.0	-	-	
A5IT52	xseA	Exodeoxyribonuclease 7 large subunit (EC 3.1.11.6)	(+)4.9	-	-	-	-	Mismatch repair
Respiration;								
A5IUP9	atpG	ATP synthase gamma chain	-	(-)1.6	-	-	(-)1.8	Bacterial secretion system; Protein export
A5IUQ0	atpA	ATP synthase subunit alpha (EC 3.6.3.14)	-	(-)1.8	-	-	-	Oxidative phosphorylation; Photosynthesis
A5IUQ1	atpH	ATP synthase subunit delta	-	(+)1.6	-	-	-	Oxidative phosphorylation; Photosynthesis
A5IRX3	SaurJH9_1146	Cytochrome bd ubiquinol oxidase, subunit I	ND	ND	-	(+)1.9	-	Two-component system; Oxidative phosphorylation
A5IV90	SaurJH9_2333	Formate dehydrogenase, alpha subunit	-	ND	ND	(+)1.7	ND	

Cell Division and Cell Cycle;								
A5ISB9	smc	Chromosome partition protein Smc	-	-	-	(+)1.6	-	
A5IS88	carB	Carbamoyl-phosphate synthase large chain (EC 6.3.5.5)	(-)2.5	(-)2.3	-	(-)2.2	(-)2.5	Alanine, aspartate and glutamate metabolism; Pyrimidine metabolism
A5IS87	carA	Carbamoyl-phosphate synthase small chain (EC 6.3.5.5)	ND	ND	(-)2.4	-	ND	Alanine, aspartate and glutamate metabolism; Pyrimidine metabolism
Regulation and Cell signaling;								
A5IVW6	SaurJH9_2562	Pyruvate oxidase (EC 1.2.3.3)	-	-	(+)1.7	(+)2.0	-	Pyruvate metabolism
A6QHH6	relA	GTP pyrophosphokinase	(+)4.0 (Induction)	ND	ND	ND	ND	Purine metabolism
Not assigned								
E5QTK7	sdrD	Serine-aspartate repeat-containing protein D	(-)24.3	-	(-)9.2	(-)2.0	-	
E5QWJ9	HMPREF0772_10696	LPXTG-motif cell wall anchor domain protein	(-)44.1	(-)9.1	(-)2.7 (Repression)	(-)22.8	(-)5.7	
A5ISU2	SaurJH9_1471	Uncharacterized protein	(-)15.2	(-)5.2	-	-	-	
A5IRR5	SaurJH9_1087	Uncharacterized protein-like protein	(-)7.5	(-)5.9	-	(-)3.3	(-)3.0	
E5QSI9	sle	N-acetylmuramoyl-L-alanine amidase Sle1 (EC 3.5.1.28)	(-)7.3	(-)3.5	-	(-)2.7	-	
A5IVG3	SaurJH9_2406	Pyridoxamine 5'-phosphate oxidase-related, FMN-binding	(-)6.2	-	-	-	-	
A5IQZ6	SaurJH9_0817	Cold-shock DNA-binding protein family	(-)5.1	(-)2.9	-	-	(-)6.5	
A5IRW9	SaurJH9_1142	Uncharacterized protein	(-)4.4	(-)2.7	-	(+)9.0	-	
A5IQG7	SaurJH9_0636	Uncharacterized protein	(-)4.1	(-)5.0	-	-	(-)3.9	
A7WYP8	spoVG	Putative septation protein SpoVG	(-)3.5	(-)2.5	-	-	-	
A5ITE0	SaurJH9_1672	UPF0473 protein SaurJH9_1672	(-)3.4	(-)2.6	-	-	-	
A5IV12	infA	Translation initiation factor IF-1	(-)3.3	(-)2.2	-	-	-	
A5IU42	SaurJH9_1930	Uncharacterized protein	(-)3.1	-	-	(-)2.1	-	
A5IST4	SaurJH9_1463	Cold-shock DNA-binding protein family	(-)3.0	(-)1.6	-	-	-	
D8HHN4	SAOV_1002c	Probable exported protein	(-)3.0	(-)4.1	-	-	(-)3.1	
A5ITK2	SaurJH9_1735	NUDIX hydrolase	(-)3.0	(-)2.5	-	-	-	
A5IVZ4	isaA	Transglycosylase domain protein, propable transglycosylase IsaA	(-)2.7	(-)10.2	-	(-)6.1	(-)2.3	
A5IRB4	SaurJH9_0936	Nitrogen-fixing NifU domain protein	(-)2.6	(-)2.1	-	-	(-)1.8	
A5IQA5	hchA	Molecular chaperone Hsp31 and glyoxalase 3 (EC 4.2.1.130)	(-)2.3	-	-	-	-	
A5IVM3	SaurJH9_2469	NAD-dependent epimerase/dehydratase	(-)2.3	-	-	-	-	
E5QSP5	HMPREF0772_11290	Glucosamine-6-phosphate isomerase family protein	(-)2.1	(-)1.6	-	-	-	
A5ISP4	SaurJH9_1423	Cell envelope-related transcriptional attenuator	(-)2.0	(-)2.0	-	-	(-)2.3	
E5QT14	HMPREF0772_10107	Uncharacterized protein	(-)2.0	-	-	(-)2.4	-	
A5IU10	SaurJH9_1898	UPF0342 protein SaurJH9_1898	(-)1.9	(-)1.9	-	-	-	
A5ITQ1	tyrS	TyrosineNDtRNA ligase (EC 6.1.1.1)	(-)1.8	-	-	-	-	Aminoacyl-tRNA biosynthesis
A5IUX1	SaurJH9_2213	Uncharacterized protein	(-)1.8	-	-	-	-	
E5QY31	HMPREF0772_12121	Uncharacterized protein	(-)1.8	-	-	-	-	
A6U1G3	SaurJH1_1431	UPF0154 protein SaurJH1_1431	(-)1.8	(-)2.2	(-)1.8	-	-	
A5ISE3	frr	Ribosome-recycling factor	(-)1.7	-	-	-	-	
A5ITI8	valS	ValineNDtRNA ligase (EC 6.1.1.9)	(-)1.7	-	-	-	-	Aminoacyl-tRNA biosynthesis
A5ISV1	SaurJH9_1480	Carboxyl-terminal protease (EC 3.4.21.102)	(-)1.7	(-)5.4	(+)2.2	-	(-)4.6	
A6QHS1	NWMN_1631	Uncharacterized protein	(-)1.7	(-)1.8	-	-		
A5IU68	gatC	Aspartyl/glutamyl-tRNA(Asn/Gln) amidotransferase subunit C (EC 6.3.5.-)	(-)1.6	(-)1.5	-	-		Aminoacyl-tRNA biosynthesis
A6QDQ0	lytM	Peptidoglycan hydrolase	(-)1.5	(-)2.1	-	(-)23.9 (Repression)	(-)2.2	
A5IRE9	SaurJH9_0971	Cof-like hydrolase	(+)1.5	-	-	-	-	
A5IPC5	SaurJH9_0241	Alcohol dehydrogenase GroES domain protein	(+)1.5	-	-	-	-	Tyrosine metabolism; Fatty acid metabolism; Glycolysis / Gluconeogenesis
E5QTL6	HMPREF0772_12636	NAD dependent epimerase/dehydratase family protein	(+)1.6	-	-	-	-	Amino sugar and nucleotide sugar metabolism; Galactose metabolism
Q6GCE3	SAS0305	Putative reductase	(+)1.6	-	-	-	-	
A5ITF5	hisS	HistidineNDtRNA ligase (EC 6.1.1.21)	(+)1.7	(+)1.8	-	-	-	Aminoacyl-tRNA biosynthesis
A5IRZ0	SaurJH9_1163	Uncharacterized protein	(+)1.8	ND	ND	-	-	
A5ISB0	SaurJH9_1284	Uncharacterized protein	(+)1.8	-	-	-	-	
Q2FDD7	repA	Replication initiator protein	(+)1.9	-	(+)2.0	(+)1.6	-	
A5IPT5	SaurJH9_0402	Acetyl-CoA acetyltransferase (EC 2.3.1.9)	(+)1.9	-	-	-	-	Two-component system; Valine, leucine and isoleucine degradation; Tryptophan metabolism; Fatty acid metabolism; Carbon fixation pathways in prokaryotes; Lysine degradation; Propanoate metabolism; Pyruvate metabolism
A5ITQ0	SaurJH9_1783	2-alkenal reductase (EC 1.3.1.74)	(+)1.9	-	(+)2.0	-	-	

E5QVA9	HMPREF0772_11628	Peptidase T-like protein	(+)1.9	(+)2.3	-	(+)1.8	-	
A8Z438	USA300HOU_1396	Glycosyltransferase (EC 2.4.1.-) group 1 family protein	(+)2.0	-	-	-	-	
A5IT20	SaurJH9_1549	Two component transcriptional regulator, winged helix family	(+)2.0	-	-	-	-	Two-component system
A5IT05	cmk	Cytidylate kinase (CK) (EC 2.7.4.25)	(+)2.0	(+)1.6	-	-	(+)1.7	Pyrimidine metabolism
A8Z0M1	USA300HOU_0269	Glycosyltransferase (EC 2.4.1.-) group 2 family protein	(+)2.0	-	-	-	-	
D9RIN2	PepF	Oligoendopeptidase F	(+)2.0	-	-	-	-	
A6QGV7	NWMN_1317	Uncharacterized protein	(+)2.1	(+)1.8	(+)1.8	(+)1.5	-	
A5IT91	SaurJH9_1621	Putative pyruvate, phosphate dikinase regulatory protein (EC 2.7.11.32) (EC 2.7.4.27)	(+)2.1	-	-	(+)2.8	-	
A5ITD7	SaurJH9_1669	Peptidase U32	(+)2.2	-	-	-	-	Epithelial cell signaling in Helicobacter pylori infection
A5IUN6	yidC	Membrane protein insertase YidC	(+)2.2	-	-	-	-	
A5IT80	pbp3	Penicillin-binding protein 3, transpeptidase	(+)2.3	-	-	-	-	Peptidoglycan biosynthesis
A5IUM8	murF	UDP-N-acetylmuramoyl-tripeptideNDD-alanyl-D-alanine ligase (EC 6.3.2.10)	(+)2.3	(+)1.6	-	-	-	Lysine biosynthesis; Peptidoglycan biosynthesis
A5IT37	SaurJH9_1566	Glyoxalase/bleomycin resistance protein/dioxygenase	(+)2.4	-	-	-	(+)2.9	Pyruvate metabolism
A5IRH7	SaurJH9_0999	Oligoendopeptidase F	(+)2.6	(+)2.0	-	-	-	
A5ITE2	SaurJH9_1674	UPF0297 protein SaurJH9_1674	(+)2.6	-	-	-	-	
E5QRM0	HMPREF0772_11165	Nitroreductase family protein	(+)2.6	-	-	-	-	
E5QY80	folD	Bifunctional protein FolD	(+)2.7	(+)1.6	-	-	-	Carbon fixation pathways in prokaryotes
Q2FDA8	SAUSA300_pUSA030030	Uncharacterized protein	(+)2.9	-	-	-	-	
A5IQA9	SaurJH9_0578	HAD-superfamily hydrolase, subfamily IA, variant 1	(+)3.0	-	-	-	(+)2.9	Glyoxylate and dicarboxylate metabolism
A5IQ95	SaurJH9_0564	16S rRNA m(2)G 1207 methyltransferase (EC 2.1.1.171)	(+)3.2	-	-	-	-	
A5IW71	SaurJH9_2668	CHAP domain containing protein	(+)3.2	ND	-	-	-	
A5IU50	SaurJH9_1938	Two component transcriptional regulator, LuxR family	(+)3.7	ND	(+)3.0	-	-	Two-component system
A5ITT5	SaurJH9_1818	Alpha/beta hydrolase fold	(+)3.7	ND	-	-	-	
A5IU07	SaurJH9_1895	Metal dependent phosphohydrolase	(+)4.0	ND	-	-	-	
A5IUJ3	SaurJH9_2084	ABC transporter related	(+)4.5	ND	ND	-	-	
E5QVV1	aroA	3-phosphoshikimate 1-carboxyvinyltransferase (EC 2.5.1.19)	(+)4.6	ND	ND	-	-	Phenylalanine, tyrosine and tryptophan biosynthesis
Q2FDA7	SAUSA300_pUSA030031	LtrC-like protein	(+)4.6	-	-	-	-	
A5INS8	ermA	rRNA (Adenine-N(6)-)-methyltransferase	(+)4.7	(+)4.2	-	-	(+)7.4	
A5ISV4	msrB	Peptide methionine sulfoxide reductase MsrB (EC 1.8.4.12)	(+)4.8	-	(+)1.8	-	-	
A5ISH1	SaurJH9_1347	Uncharacterized protein	(+)5.7	-	-	-	-	
A6QED6	tmk	Thymidylate kinase (EC 2.7.4.9) (dTMP kinase)	(+)6.3	(+)4.8	-	(+)2.2	(+)5.6	Pyrimidine metabolism
A8Z1L4	purH	Bifunctional purine biosynthesis protein PurH	(+)9.8	ND	ND	-	-	One carbon pool by folate; Purine metabolism
D9RDE0	SAA6159_00214	ABC superfamily ATP binding cassette transporter, binding protein	(+)3.9 (Induction)	ND	-	-	-	
A5IU99	SaurJH9_1988	ABC transporter related	(+)12.2 (Induction)	ND	(+)3.3	-	-	ABC transporters
A5ISN5	SaurJH9_1414	HesB/YadR/YfhF-family protein	(+)4.0 (Induction)	ND	-	-	-	
E5QUJ2	HMPREF0772_11518	Luciferase family oxidoreductase, FMN-dependent, PP_0088 family (EC 1.-.-.-)	(+)5.8 (Induction)	(+)4.0 (Induction)	ND	(+)2.7	ND	
A5ISV5	msrA	Peptide methionine sulfoxide reductase MsrA (EC 1.8.4.11)	(+)8.5 (Induction)	ND	(+)8.0	ND	ND	
A5ISH4	SaurJH9_1350	Pyruvate ferredoxin/flavodoxin oxidoreductase, beta subunit	(+)4.0 (Induction)	ND	ND	ND	ND	Citrate cycle (TCA cycle); Carbon fixation pathways in prokaryotes
A5ISS3	SaurJH9_1452	Uncharacterized protein-like protein	(+)6.1 (Induction)	(+)3.8 (Induction)	ND	ND	ND	
Q6GGX3	ebh SAR1447	Extracellular matrix-binding protein ebh (ECM-binding protein homolog)	(-)27.7 (Repression)	(-)4.8	ND	ND	(-10.7) (Repression)	
A5IUP1	sceD SaurJH9_2132	Transglycosylase domain protein,probable transglycosylase SceD	(-)22.9 (Repression)	(-)12.5 (Repression)	ND	-	ND	
A5IQJ6	SaurJH9_0666	ABC transporter related	-	-	(+)1.9	-	-	
A5IWE3	SaurJH9_2761	Aminoglycoside phosphotransferase	-	-	-	(+)2.0	-	
A8Z3P8	USA300HOU_1149	Bifunctional phosphopantothenoylcysteine decarboxylase/phosphopantothenateNDcysteine ligase (EC 4.1.1.36) (EC 6.3.2.5)	-	-	(+)1.5	(+)2.0	-	Pantothenate and CoA biosynthesis
E5QR16	HMPREF0772_11041	Cof-like hydrolase	-	-	-	(+)2.1	-	
D3ETT8	SA2981_0533	Deoxyadenosine kinase / Deoxyguanosine kinase (EC 2.7.1.113) (EC 2.7.1.76)	-	(+)1.9	-	-	-	
A5ISX2	SaurJH9_1501	Dynamin family protein	-	-	-	(+)2.3	-	
E5QTK9	azo	FMN-dependent NADPH-azoreductase (EC 1.7.-.-)	-	-	(+)1.8	-	-	
A5ITR7	SaurJH9_1800	Glutamyl aminopeptidase (EC 3.4.11.7)	ND	(+)7.2	-	-	-	
E5QSC4	ltaS	Glycerol phosphate lipoteichoic acid synthase (EC 2.7.8.-)	-	(-)14.7	-	(-)2.5	-	
A5ISY0	SaurJH9_1509	Glycosyl transferase, family 51	-	(-)1.5	-	-	-	Peptidoglycan biosynthesis
D1GTX1	isaB	Immunodominant antigen B	-	-	(+)2.4	-	-	

A7WWT7	aadD	Kanamycin nucleotidyltransferase	-	-	(+)1.7	-	-	
A5IRC0	SaurJH9_0942	Leucyl aminopeptidase (EC 3.4.11.1)	-	-	(+)2.2	-	-	
A7WZ39	SAHV_0592	Mercuric reductase homologue	-	(+)3.3	-	-	-	
A5ISH2	SaurJH9_1348	Metallophosphoesterase	-	(+)3.2	-	-	-	
A5IV82	SaurJH9_2325	NAD/NADP octopine/nopaline dehydrogenase	-	-	-	(+)1.7	-	
A5IVV0	SaurJH9_2546	Nitroreductase	-	-	-	(+)1.7	-	
A5IQU7	pepT	Peptidase T (EC 3.4.11.4)	-	-	(+)2.6	-	-	
Q2YYU5	SAB2172	Probable secretory antigen	-	(-)3.2	-	-	(-)2.5	
D8HDH4	spa	Protein A	ND	ND	(-)4.5 (Repression)	ND	ND	
E5QWF6	ptsG	PTS system, glucose-specific IIBC component (EC 2.7.1.69)	-	-	-2.6	(+)1.8	-1.8	
Q2FDC9	SAUSA300_pUSA030009	Putative regulator of transfer genes ArtA	-	-	(+)1.9	-	-	
D8HC44	SAOV_1724	Putative uncharacterized protein	-	-	(-)2.4	-	-	
Q2YXM8	SAB1151	Pyruvate ferredoxin oxidoreductase alpha chain (EC 1.2.7.1)	-	-	(+)3.3	(+)3.3	-	Citrate cycle (TCA cycle); Carbon fixation pathways in prokaryotes
A5IT81	SaurJH9_1611	Superoxide dismutase (EC 1.15.1.1)	-	(+)1.7	-	-	-	
E5QRW8	HMPREF0772_12410	TIGR01777 family protein	-	-	-	(+)1.7	-	
A5IQP0	SaurJH9_0710	Transcriptional regulator, MarR family	-	-	-	-1.5	-	
E5QXI6	HMPREF0772_10873	Uncharacterized protein	-	-	(+)3.2	(+)2.7	-	
A5ITC2	SaurJH9_1652	Uncharacterized protein	-	-	(+)1.7		-	
A5IUW2	SaurJH9_2204	Uncharacterized protein	-	-	-	-1.8	-	
A5IVL3	SaurJH9_2459	Uncharacterized protein	-	-	-	(+)2.6	-	
A5IU58	SaurJH9_1946	Uncharacterized protein	-	(-)1.9	-	-	(-)2.7	
E5QVA7	HMPREF0772_11626	Uncharacterized protein	-	ND	-	-	(+)2.1	
A5IPW4	SaurJH9_0432	Uncharacterized protein	-	(+)1.9	-	-	(+)1.8	
A5ITS4	SaurJH9_1807	Uncharacterized protein	-	-	-	-	(+)1.7	
A5IVI0	nirB	Assimilatory nitrite reductase (NAD(P)H) large subunit (EC 1.7.1.4)	(-)35.8	(-)6.0	-	-	(-)26.7	Nitrogen metabolism
A5IUL2	SaurJH9_2103	Stage II sporulation protein	(-)1.6	-	-	-	-	
A6QGK7	glnA	Glutamine synthetase (EC 6.3.1.2)	(-)1.6	-	-	-	-	
A5ISU1	SaurJH9_1470	ATPase associated with various cellular activities, AAA_5	(+)1.5	(-)1.8	-	-	(-)1.8	
A6QIE7	ppaC	Probable manganese-dependent inorganic pyrophosphatase (EC 3.6.1.1)	(+)2.0	(+)2.0	-	-	-	
A5IWF1	SaurJH9_2749	Beta-lactamase (EC 3.5.2.6)	(+)4.0 (Induction)	(+)16.8 (Induction)	ND	(+)64.6 (Induction)	ND	
A6QJN7	narG	Nitrate reductase, alpha subunit	(-)73.4 (Repression)	(-)6.4	-	-	(-)28.4	Nitrogen metabolism
A5IVH7	narH	Respiratory nitrate reductase beta subunit	(-)21.0 (Repression)	(-)7.5	-	-	(-)8.1 (Repression)	Nitrogen metabolism
A5IQI6	SaurJH9_0656	ABC transporter related	ND	ND	-	(-)16.6 (Repression)	-	ABC transporters
A5IUK9	SaurJH9_2100	RNA binding S1 domain protein	-	-	(-)1.7	-	-	
D2N839	SAPIG1763	Universal stress protein family	-	-	(+)2.2	(+)1.8	-	
A6QJP6	NWMN_2306	Zn-binding lipoprotein adcA-like protein	-	-		(+)2.3	-	
A5ITN0	SaurJH9_1763	UPF0173 metal-dependent hydrolase SaurJH9_1763	-	-	(+)2.3	-	-	
A5IQE1	SaurJH9_0610	UPF0447 protein SaurJH9_0610	-	(+)1.9	-	-	-	
Q2FZ98	SAOUHSC_01139	UPF0747 protein SAOUHSC_01139/SAOUHSC_01140/SAOUHSC_01141	-	(+)8.7	-	-	-	

Note: The fold changes with (+) stand for up-regulated proteins and those with (-) stand for down-regulated proteins. The symbol of "–" stands for non-differentially expressed protein and "ND" stands for non-detected protein. The proteins which cannot be detected in the blank but detected in the drug treatment group are considered as "induction"; the proteins which can be detected in the blank but cannot be detected in the drug treatment group are considered as "repression". For the induction or repression proteins, the spectral counts are set to 1 for non-detected proteins to calculate fold changes.

Table S2. Minimum inhibitory concentrations (MIC) of oxacillin in the presence of SIPI-8294 and erythromycin against MRSA (ATCC43300), and the fractional inhibitory concentration (FIC) indexes in each case.

**Compound**	**MIC of compound alone**	**Concentration (μg/mL)**	**Oxacillin MIC (μg/mL)**	**FIC index**	**Synergy**
Oxacillin	64	—	—	—	—
SIPI-8294	64	4	8	0.19	Yes
		8	0.25	0.13	
		16	0.0625	0.25	
Erythromycin	>512	4	64	1.01	No
		32	64	1.06	

Note: FIC ≦ 0.5 indicates synergy
